# A patent review of von Hippel-Lindau (VHL)-recruiting chemical matter: E3 ligase ligands for PROTACs and targeted protein degradation (2019–present)

**DOI:** 10.1080/13543776.2024.2446232

**Published:** 2025-01-21

**Authors:** Aina Urbina, Alex J. Hallatt, Jack Robertson, Alessio Ciulli

**Affiliations:** Centre for Targeted Protein Degradation, School of Life Sciences, University of Dundee, Dundee, UK

**Keywords:** PROTACs, von Hippel-Lindau, E3 ligase ligand, targeted protein degradation, structure-activity relationship, bifunctional molecule, hydroxyproline, bioisosteres

## Abstract

**Introduction:**

The von Hippel-Lindau (VHL) E3 ubiquitin ligase has seen extensive research due to its involvement in the ubiquitin proteasome system and role as a tumor suppressor within the hypoxia signaling pathway. VHL has become an attractive target for proteolysis targeting chimeras (PROTACs), bifunctional molecules that can induce degradation of neo-substrate proteins. The development of VHL inhibitors and PROTACs has seen rapid development since disclosure of the first non-peptidic VHL ligand (2012).

**Areas covered:**

Due to the demand for more diverse and sophisticated VHL ligands that can be applied to PROTACs, the number of patents disclosed has risen significantly in the past 5 years. Herein, the wide range of VHL modifications that have been patented since 2019 is covered. Specifically, any new or unique chemical modification to established VHL ligands or PROTACs will be discussed.

**Expert opinion:**

The VHL chemical space continues to expand within the patent literature. There are exciting new modifications that can enhance the physiochemical properties of VHL PROTACs and other alterations can improve the affinity of the VHL ligand itself. Further optimization of the VHL chemical space will no doubt lead to the development of more VHL-based therapies and clinical candidates.

## 1. Introduction

### 1.1. VHL function

The von Hippel-Lindau (*vhl*) gene was first discovered in 1993 by Lerman *et al*. through a positional cloning campaign [1]. The von Hippel-Lindau (VHL) protein itself is a substrate recognition subunit of the Cullin 2 RING-VHL E3 ligase (CRL2^VHL^) that functions as part of the ubiquitin-proteasome system (UPS) [2,3]. The UPS is the cellular machinery responsible for the degradation of intracellular protein targets. The UPS cascade functions where a target protein becomes tagged with the relatively small protein ubiquitin via consecutive action of E1 ubiquitin activating enzymes, E2 ubiquitin conjugating enzymes, and ultimately the E3 ubiquitin ligases [4]. The ubiquitin is covalently tagged to the target protein as mono- and poly-ubiquitin chains and acts as a recognition group for the 26S proteasome that can degrade the ubiquitinated protein [4,5]. VHL has been extensively studied due to its involvement in the binding and subsequent ubiquitination/degradation of the hypoxia-inducible factor alpha-subunit (HIF-α) [6].

The primary role of VHL is as a tumor suppressor which can lead to degradation of HIFs, primarily HIF-1α and HIF-2α [7]. HIFs are transcription factors that transactivate genes that encode for proteins that play an important role in how cells adapt to hypoxic environments [8–10]. HIF transcription factors often code for genes important in cell proliferation and angiogenesis [11,12]. The nature of HIFs means that VHL is essential to turn off certain cellular adaptations under normoxic conditions to avoid unwanted cell proliferation [13].

In a normoxic environment, two conserved proline residues of HIF-1α (Pro402 and Pro564) are hydroxylated by prolyl hydroxylase domain-containing enzymes (PHDs) [14,15]. After prolyl hydroxylation, HIF-α is recognized by VHL, ubiquitinated, then subsequently degraded by the proteasome. This prolyl hydroxylation recognition by VHL became the basis for ligand development for VHL.

### 1.2. VHL as a target

Leveraging the interaction between VHL and HIFs was first investigated by Pugh *et al*. where it was demonstrated that peptides derived from HIF-1α could be expressed intracellularly and interfere with HIF degradation [16]. Interaction of HIF-derived peptides with VHL was confirmed through a series of VHL assays.

Building on the work by Pugh *et al*., the concept of proteolysis targeting chimera (PROTACs) was developed through work in the Deshaies and Crews labs [17]. PROTACs were proposed as bifunctional compounds that can bind to both an E3 ligase and a different protein target to trigger UPS-mediated degradation of the target protein. Initial VHL-binding PROTAC species were peptide-based and demonstrated the ability to degrade green fluorescent protein (GFP) fusions with FKBP12 and androgen receptor upon microinjection into cells or via addition of cell-penetrating peptides [18].

**Figure 1.**
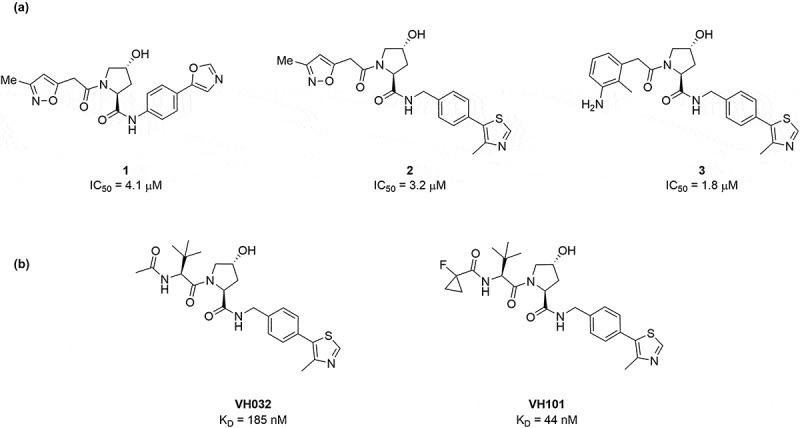
Development of VHL ligands from micromolar to nanomolar K_D_. (a) Initial ligands **1-3** developed by Buckley et al. IC_50_ values to VHL calculated by isothermal titration calorimetry (ITC). (b) Potent VHL ligands developed by Ciulli et al. K_D_ measured using fluorescence polarization (FP).

The potential of targeting VHL and the UPS to remove undesirable proteins led to the development of non-peptidic small-molecule VHL ligands by Crews and Ciulli labs [19]. First-generation compounds were built around the (2*S*,4*R*)-4-hydroxyprolinecore, also known as *trans*-hydroxyproline or Hyp, to recapitulate the critical ‘hot-spot’ of protein-protein interactions between VHL and HIF-1α. This Hyp scaffold initially led to the development of compounds **1**, **2**, and **3** with modest disruption of the VHL:HIF interaction, with IC_50_ values from 4.1 µM to 1.8 µM (Figure 1(a)) [20,21]. Structure-guided design of ligands **1-3** gave rise to the established VHL ligands **VH032** and **VH101** with impressive binding affinities to VHL, with K_D_ values of 185 nM and 44 nM respectively (Figure 1(b)) [22,23]. The optimized ligands contained a phenyl group connected to a methyl thiazole moiety on the right-hand side of the Hyp core with a *tert*-leucine group on the left-hand side of the Hyp core and, in the case of **VH101**, a capping strategy at the N-terminus invoking a fluoro-cyclopropyl group.

### 1.3. VHL engagement in PROTACs

**Figure 2.**
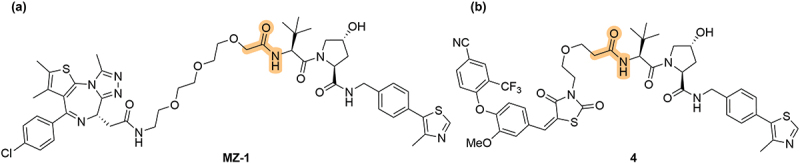
The first disclosed VHL ligand-containing PROTACs. (a) BRD4 targeting PROTAC **MZ-1**. (b) ER PROTAC **4**.

With the identification of potent VHL ligands, the opportunities to apply them to PROTAC technology rapidly expanded the variety of VHL chemical matter. The first set of VHL-based PROTACs were degraders of the bromodomain-containing protein 4 (BRD4) linked from the N-terminal amide group of **VH032**, published from the Ciulli lab, with **MZ-1** proving the most effective PROTAC (Figure 2(a)) [24]. The estrogen-receptor (ER) PROTAC **4** was subsequently published by the Crews-GSK collaboration, also linking the from N-terminal amide of the VHL ligand (Figure 2(b)) [25].

**Figure 3.**
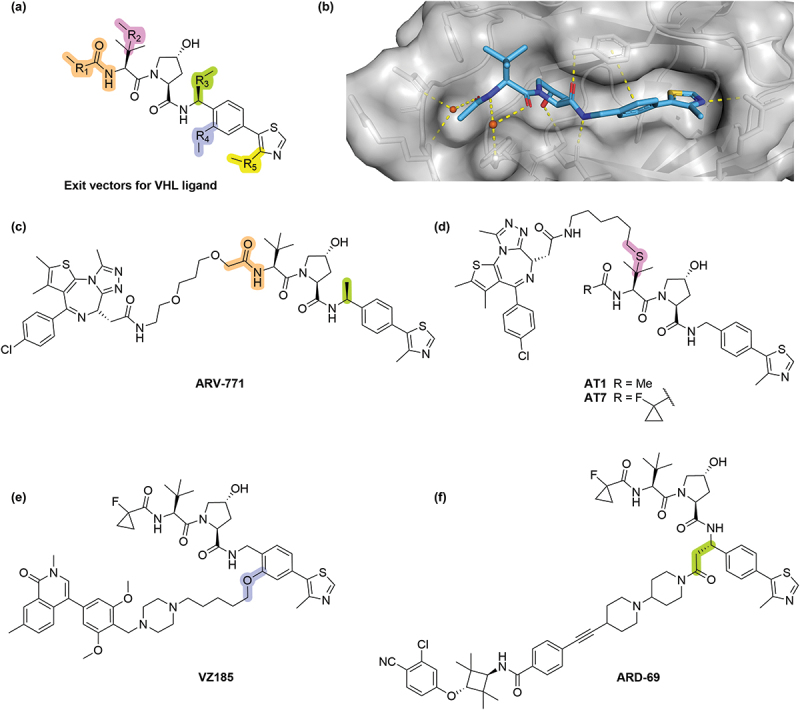
Early PROTACs exemplifying the various exit vectors on the VHL ligand. (a) The five primary exit vector positions within the **VH032** ligand. (b) Cocrystal structure of **VH032** bound to VCB (PDB 4W9H). (c, d) BRD2/3/4 targeting PROTACs **ARV-771**, **AT1** and **AT7**. (e) BRD7/9 targeting PROTAC **VZ185**. (f) AR targeting PROTAC **ARD-69**.

Further PROTAC development in the years after **MZ-1** has seen various linker tethering sites identified within the **VH032** scaffold (Figure 3(a)) [26,27]. The structure-guided exploitation of these linker attachment vectors was enabled by the rapid rise of ligand-bound crystal structures of VHL, which is routinely expressed and crystallized in complex with the adapter proteins Elongin B and Elongin C (referred to collectively as the VCB complex). The N-terminal amide group (R_1_) has been extensively used as a linker handle, with **ARV-771** being another representative PROTAC built up from this exit vector. Originally reported by Arvinas Operations, Inc. (Arvinas) in the development of this pan-BET (Bromodomain and Extra Terminal protein) degrader in 2016, **ARV-771** features the stereoselective introduction of a methyl group in the benzylic position that is a popular modification in VHL ligands, offering an improved inhibitory potency of VHL [28–30]. The *tert*-leucine group has also been utilised as an exit vector (R_2_), with its replacement for the isosteric penicillamine suitable for linker attachment via thioether bond formation. This was first demonstrated in 2017 by the Ciulli lab with the development of the BET PROTAC **AT1**, which selectively degraded BRD4 over BET family members BRD2 and BRD3, and later analogue **AT7** which incorporated the beneficial fluoro-cyclopropyl group to cap the N-terminal amide [31,32]. Initially explored in the development of HaloPROTACs, the phenyl ring of the VHL has been exploited as an exit vector via a phenol ether (R_4_) [33,34]. A representative example of the utilization of this exit vector in PROTAC synthesis was the BRD7/BRD9 degrader **VZ185** published in 2019 [35]. The tolerance of the chiral methyl at the benzylic position led to postulation that this solvent-exposed vector (R_3_) could be exploited as a handle for linker attachment, first demonstrated with the Androgen Receptor (AR)-targeting PROTAC **ARD-69** from the Wang lab [36]. Similarly, the solvent-exposed nature of the methyl substituent on the thiazole group has made this another sought-after exit vector from the VHL ligand (R_5_), although this milestone was achieved post-2019 and so will be discussed in the relevant section herein (see section 6).

### 1.4. Scope of this review

The modification of VHL ligands as inhibitors and in PROTACs has progressed rapidly in recent years, with many pharmaceutical companies seeking to expand the field of targeted protein degradation (TPD) and four known VHL-based degraders are currently in clinical trials (Table 1) [37–41]. Previous excellent reviews have covered the development of VHL ligands and PROTACs pre-2019 in detail [26,27,42]. Here we present the latest developments of VHL chemical matter in patent literature published between 1 January 2019 until 31 August 2024, for both PROTAC and inhibitor species. We used SciFinder to identify 132 patents which included both the *trans*-Hyp motif and the phrase ‘VHL,’ which were published in the defined date range. We used manual inspection to refine this initial dataset down to a total of 93 patents which were of value for the scope of this review.

**Table 1.** VHL-based PROTAC degraders currently in clinical trials. Only two have disclosed chemical structures, (A) **DT2216** and (B) **KT-333**.

Degrader	Company	Target	Clinical Trial	Ref
**DT2216**	**Dialectic Therapeutics**	BCL-xL	NCT04886622	[37,38]
**KT-333**	**Kymera Therapeutics**	STAT3	NCT05225584	[39]
**ASP3082**	**Astellas Pharma**	KRAS^G12D^	NCT05382559	[40]
**PRT3789**	**Prelude Therapeutics**	SMARCA2/4	NCT05639751	[41]
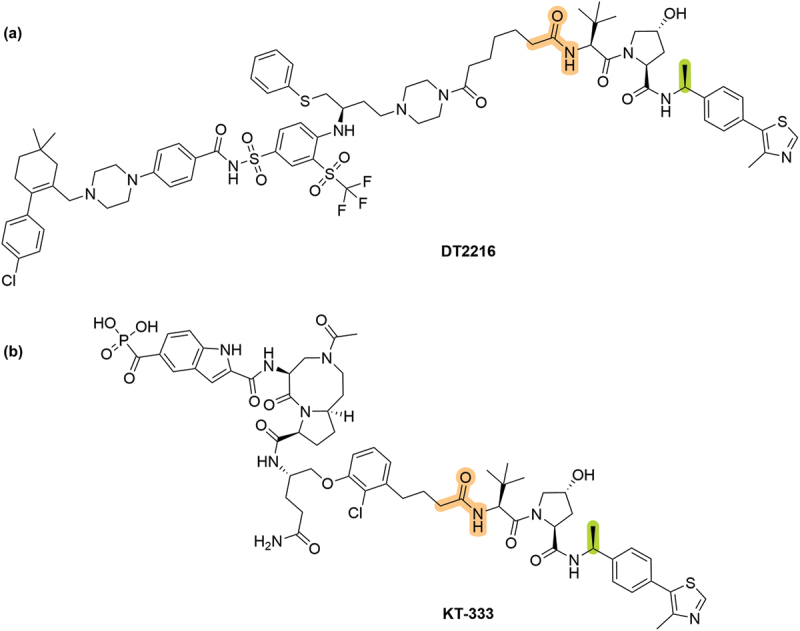				

The focus of this review will be the changes to the VHL ligand itself, rather than modifications to the protein of interest (POI) ligand or linkerology, as the scope of this would be too broad and would be better served in bespoke reviews for each target protein class, as exemplified by recent patent reviews for degraders of BRD4, epidermal growth factor receptor, ER, and SMARCA2 [43–46]. Despite this, we will still be commenting on any relevant biological data associated with the reported compounds, where available.

**Figure 4.**
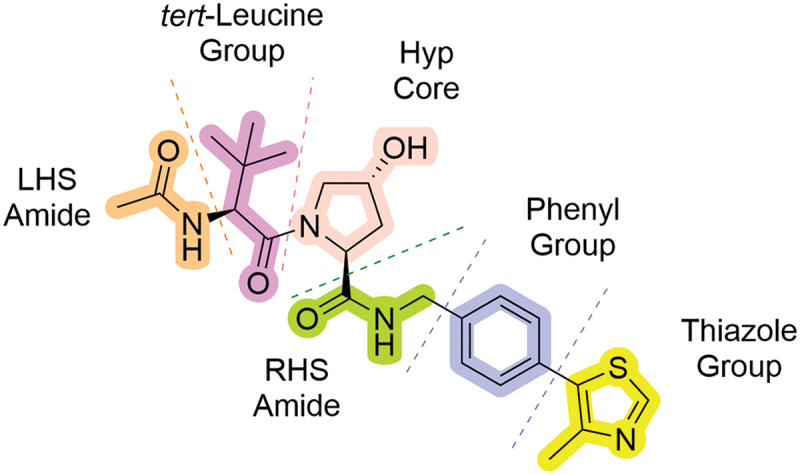
The six key parts of the VHL ligand that will be covered in this review.

Discussion of the chemical modifications will be divided into 6 sections which correspond to the different segments of the VHL ligand (Figure 4). The Hyp core connects the left-hand side (LHS), containing a terminal amide and a flanked *tert*-leucine unit, to the right-hand side (RHS), which contains a benzylamine group tethered to a thiazole ring at the 4-position.

## 2. LHS amide modifications

The peptidic nature of the LHS of the VHL ligand enabled this region of the ligand to be rapidly modified during structure–activity relationship (SAR) campaigns of early VHL inhibitors, owing in part due to the synthetic ease of terminal amide bond formation and the accessibility for expansion in this solvent-exposed region, as in the native HIF peptides. As a result, the terminal LHS amide was the first region of the VHL ligand to be exploited to conjugate to protein of interest (POI) binders [47]. Prior to 2019, modification of this position with various linear amides accounted for the vast majority of the PROTAC literature of the time, and so this kind of modification will not be covered herein.

**Figure 5.**
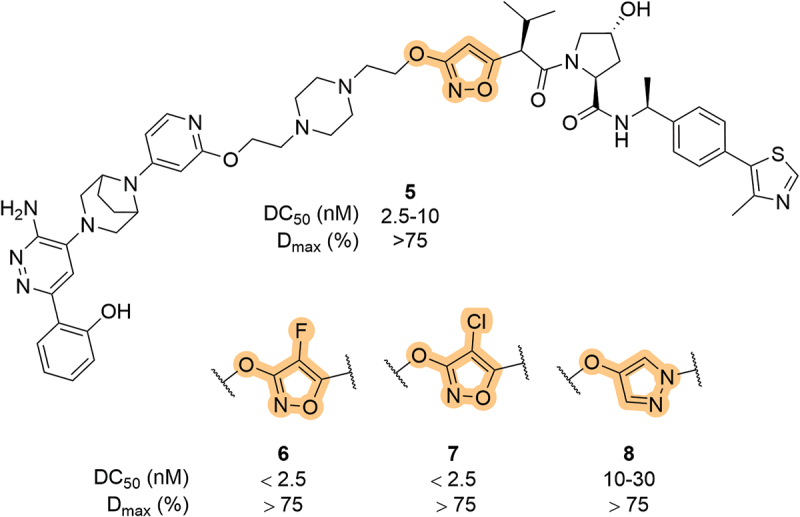
VHL-recruiting SMARCA2 degraders with heteroaryl groups in place of the LHS amide, patented by Arvinas.

In 2019, Arvinas patented a series of SMARCA2/4 degraders based on the canonical VHL scaffold [48]. Within the patented compounds, 3,5-disubstituted isoxazoles were used as an isostere for the LHS amide group, effectively removing the hydrogen bond donor. Of note, the early VHL ligands published in 2012 incorporated a methyl-isoxazole group at this exact position (Figure 1(a)) [19–21]. The 5-position connects the isoxazole to the rest of the VHL ligand, whilst the 3-position is used to install the linker, often with a heteroatom handle, as highlighted in compound **5** (Figure 5). Within this patent, they also explored the substitution of the 4-position of the isoxazole ring with fluorine (**6**) and chlorine atoms (**7**), with both showing good degradation activity. *N*-linked pyrazoles were also explored (**8**) but were shown to be less effective at inducing SMARCA2 degradation. Isoxazole, either as a terminal motif or as a LHS exit vector, have been utilized heavily in other Arvinas patents targeting other POIs, such as Kirsten rat sarcoma virus (KRAS), Tau, and rapidly accelerated fibrosarcoma (RAF) proteins [49–52]. Since 2019, the number of patents containing VHL-recruiting PROTACs that bear a LHS isoxazole motif has increased dramatically and thus it has become a staple in the repertoire of VHL ligands.

**Figure 6.**
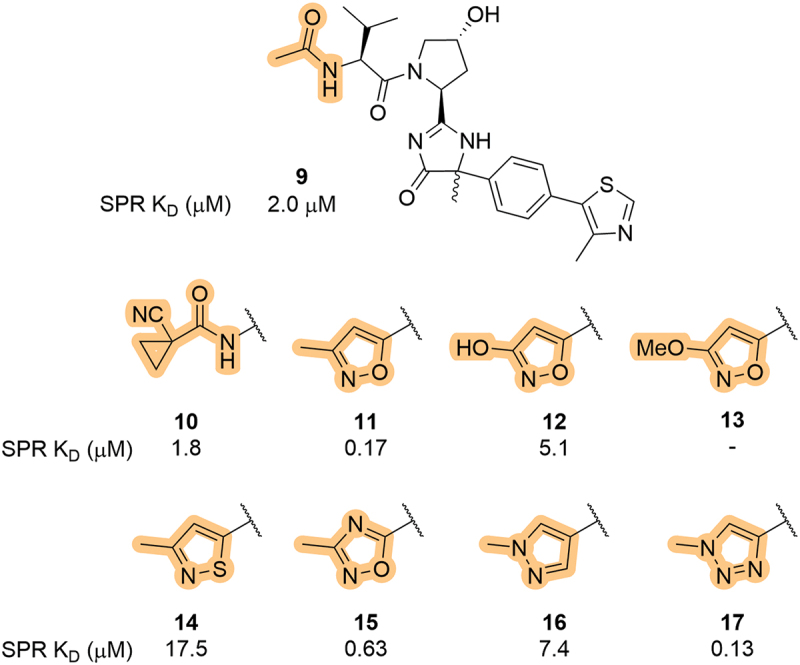
Systematic exploration of five-membered heterocycles in place of the LHS amide in VHL inhibitors, as patented by Genentech.

Also in 2019, Genentech, Inc. (Genentech) patented a series of VHL inhibitors in which the LHS terminal group was systematically explored in the context of a novel cyclization at the RHS amide region (amide mimetics of the RHS amide covered in section 4) [53]. While familiar LHS motifs were reported, such as *N*-acetyl (**9**) and cyano-cyclopropyl (**10**), a range of heteroaromatic groups were used in place of the amide moiety (Figure 6). The reported compounds were characterized using a range of assays, including fluorescence polarization (FP), bioluminescence resonance energy transfer (BRET), and surface plasmon resonance (SPR), which was used to determine binding affinity (K_D_). As previously shown in patents by Arvinas and published literature, the 3-methylisoxazole analog (**11**, K_D_ = 0.17 µM) showed a roughly 10-fold increase in binding affinity, although the 3-hydroxy analog (**12**, K_D_ = 5.1 µM) showed comparable affinity to *N*-acetyl (**9**, K_D_ = 2.0 µM). As such, the beneficial methyl group was fixed at the 3-position and various five-membered heteroaromatic groups were explored, including isothiazole (**14**), oxadiazole (**15**), pyrazole (**16**), and 1,2,3-triazole (**17**). Both the isothiazole and pyrazole showed a loss of affinity (K_D_ = 17.5 and 7.4 µM, respectively) and the oxadiazole and 1,2,3-triazole showed similar affinity (K_D_  = 0.63 and 0.13, respectively) to the parent isoxazole analog **11**.

**Figure 7.**
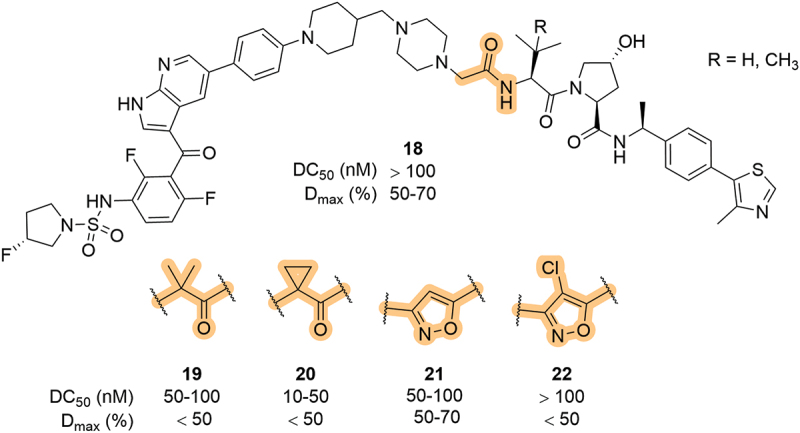
Modulation of the LHS amide in BRAF targeting degraders, patented by Arvinas.

Arvinas patented a series of degraders targeting RAF proteins, in particular BRAF V600E mutants, in 2020, which involved minor changes to the LHS amide [52]. Interestingly, the addition of methyl (**19**) and cyclopropyl (**20**) groups at the alpha position improved the DC_50_ of the compounds (50–100 and 10–50, vs >100 nM) but had a detrimental effect on the D_max_ (Figure 7). As in their other patents, Arvinas also incorporated 3,5-disubstituted isoxazoles into their BRAF degraders which led to improved degradation potencies, as exemplified by **21**, although incorporation of a chlorine atom at the 4-position of the ring (**22**) led to a significant increase in DC_50_ and reduced D_max_.

**Figure 8.**
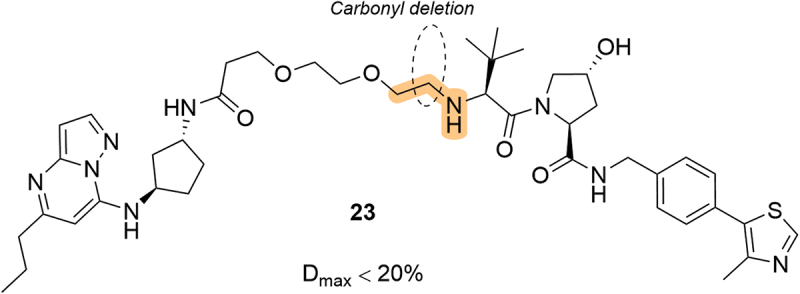
Putative CDK9 degraders bearing a LHS amino linkage, patented by Massachusetts Institute of Technology.

Massachusetts Institute of Technology (MIT) patented a curious series of putative Cyclin-dependent kinase 9 (CDK9) degraders in 2021, in which they did not use an amide functionality to attach the linker, as in the canonical way, but rather an amine linkage (**23**, Figure 8) [54]. They disclosed 8 VHL examples in total, with various length polyethylene glycol (PEG) and phenyl-based linkers, although they only displayed borderline degradation of CDK9 by western blot. They did not report any VHL-based degraders with an N-terminal amide on the VHL ligand to compare against, although they did disclose a series of Cereblon-based degraders which showed much improved efficacy. Surprisingly, a similar patent by Ikena Oncology, Inc. was published in 2022 which also described a series of putative VHL-recruiting bifunctional compounds linked by a LHS amino group, although they were unable to demonstrate any efficacy *in vitro* [55].

**Figure 9.**
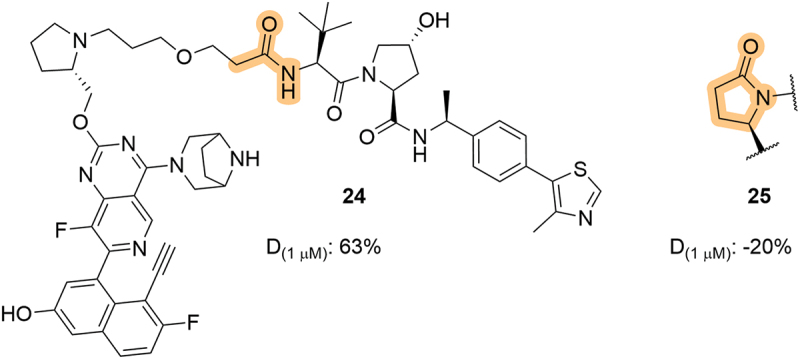
Modulation of the LHS amide in KRAS^G12D^ targeting degraders, including lactam **25**, patented by Erasca. D_(1 μM)_ = degradation achieved after compound treatment at 1 μM, where a negative value denotes stabilization of target protein levels.

In 2022, Erasca, Inc. (Erasca) patented a series of VHL-based KRAS degraders which specifically target the G12D mutation [56]. Although the LHS of the VHL ligand was generally conserved among the reported compounds, they did disclose an interesting LHS lactam compound, **25** (Figure 9). Unfortunately, cyclization of the amide-linker moiety (from the moderately active degrader **24**) to afford the corresponding lactam led to a complete loss of activity in a KRAS^G12D^ HiBit knock-in cell line, consistent with the loss of binary VHL binding due to this modification which was first reported in the SAR study of LHS capping groups by Soares *et al*. [23].

**Figure 10.**
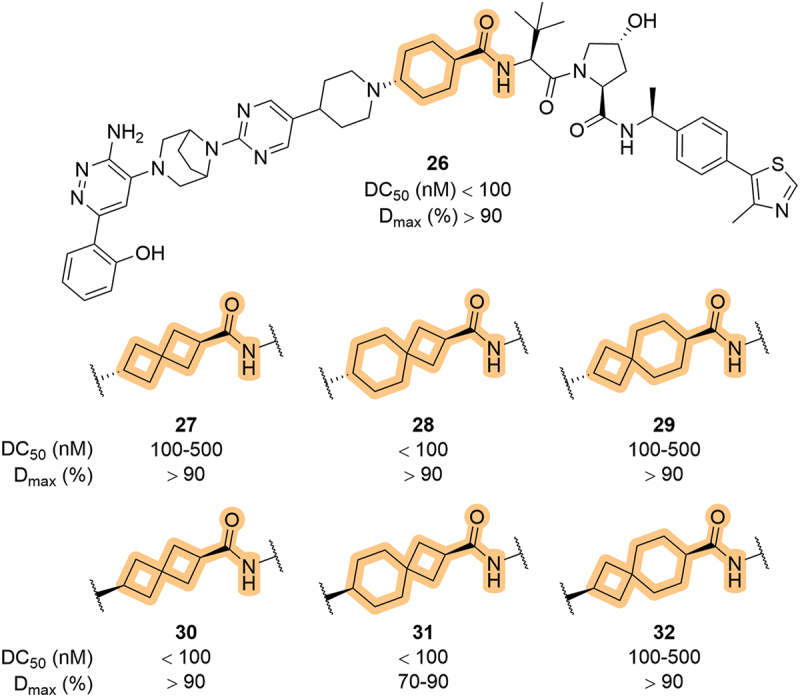
Modulation of the LHS amide to include spirocycles in dual SMARCA2/4 degraders, patented by Kymera.

In 2022, Kymera Therapeutics, Inc. (Kymera) patented a series of dual SMARCA2/4 degraders which bore spirocyclic groups at the LHS amide (Figure 10) [57]. While this modification blurs that line between VHL ligand and linker, it is potentially important to note that while many of the compounds could effectively degrade SMARCA2 levels by more than 70% (**26-32**), the compounds bearing a cyclobutyl group closest to the LHS amide were generally more potent. This use of spirocycles at the LHS part of VHL-recruiting degraders has been utilized in other patents that further target SMARCA or other protein classes [58–63].

**Figure 11.**
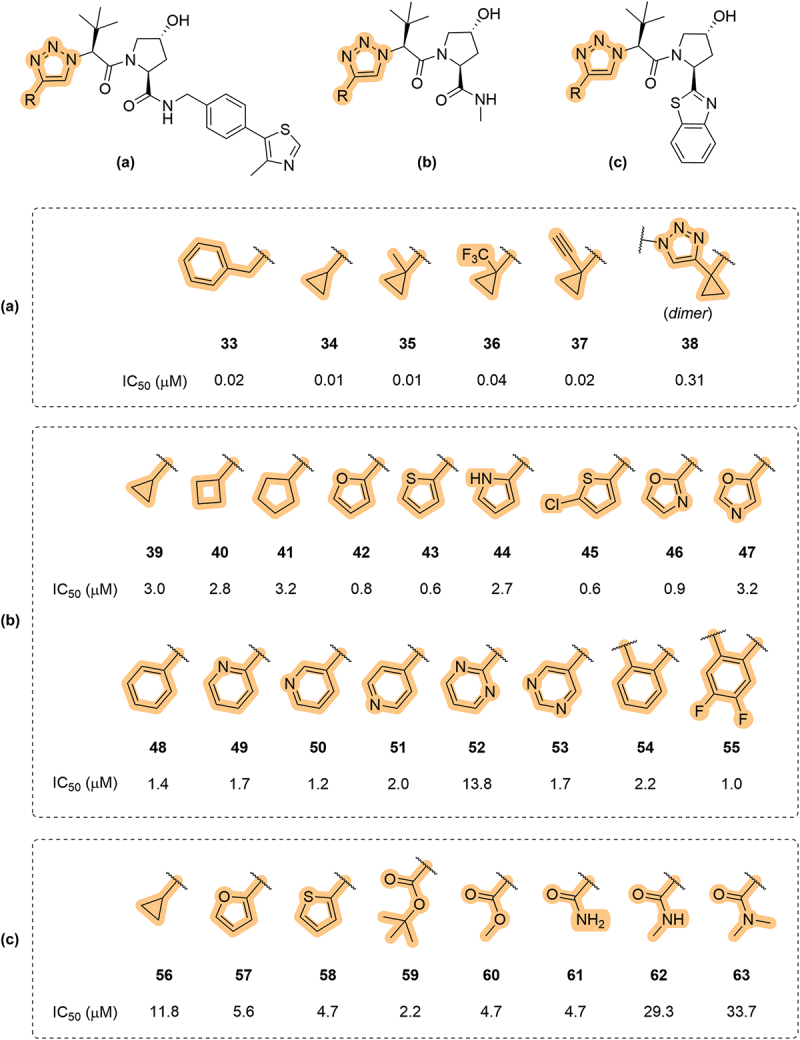
Exploration of the LHS 1,2,3-triazole with many substituents at the 4-position, for use as VHL inhibitors, as patented by Genentech. Each VHL inhibitor contained either, the full canonical (a), truncated (b) or modified (c) RHS of the VHL ligand.

Between May 2022 and November 2023, Genentech published multiple patents which disclosed a range of VHL inhibitors bearing substituted 1,2,3-triazoles in place of the LHS amide [64–67]. Each of these patents used a different RHS of the VHL ligand, but in all of them the substituent on the LHS triazole was systematically varied (Figure 11). The first patent focused on LHS modifications in the context of the full canonical **VH032** RHS (Figure 11(a)), whereas the second patent centered on a truncated form of the VHL RHS (Figure 11(b)) [64,65]. The final patent, published in 2023, replaced the entire RHS with a benzothiazole moiety (Figure 11(c)) although this interesting RHS modification is discussed in detail in section 4 [66]. In general, there was a wide tolerance for substitution at the 4-position of the triazole ring, particularly when the canonical RHS was retained (**33-38**, Figure 11(a)). Small carbocycles such as cyclopropyl (**39**), cyclobutyl (**40**) and cyclopentyl (**41**), showed cellular IC_50_ values in the low single-figure µM range. Phenyl (**48**) and pyridyl substituents (**49-50**) were also well-tolerated at the 4-position and interestingly the position of the nitrogen atom in the pyridine ring did not appear to have a notable effect on activity toward VCB. However, five-membered heteroaromatic substituents (**42-47**) gave the optimal IC_50_ values, leading to up to a 5-fold improvement relative to the carbocycle comparator (**41**). Furan (**42**) and thiophenes (**43** and **45**) were the most potent of the series, and the inclusion of additional heteroatoms, to afford oxazoles **46** and **47**, did not improve potency. Notable compounds were disclosed in the 2023 Genentech patent, which contained ester and amide substituents on the triazole ring (**59-63**). While the *tert*-butyl ester **59**, methyl ester **60** and primary amide **61** were well tolerated, both the secondary and tertiary amides (**62** and **63**, respectively) showed an almost 10-fold loss in cellular activity. This finding may suggest that installing linkers to the LHS triazole may be detrimental to efficacy if the linker is tethered to the triazole via an amide. It is also mentioned in this patent, although the data was not shown, that all of the triazole-bearing compounds exhibited superior permeability than the parent VHL ligand, **VH032**, in a Madin-Darby canine kidney cells (MDCK) permeability assay. The scope of LHS triazole modifications was taken further in a subsequent patent by Genentech, but the overall finding was that increasing steric bulk, beyond that of a single heterocycle, led to typically a > 5-fold loss of activity toward VCB [67].

**Figure 12.**
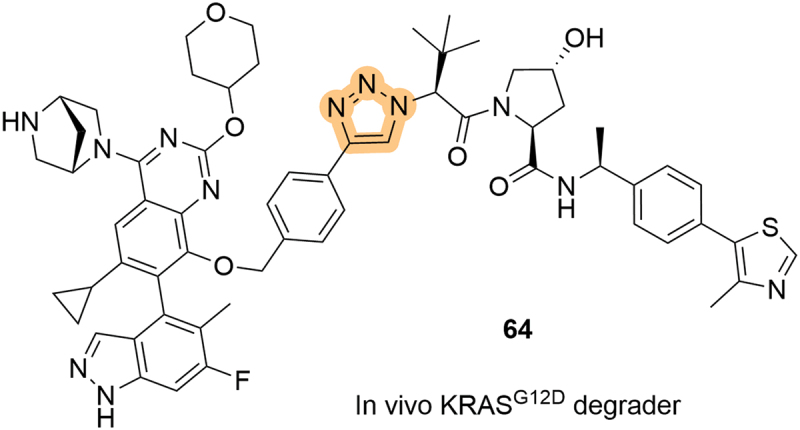
In vivo KRAS^G12D^ degrader containing a LHS 1,2,3-triazole, as patented by Astellas.

The utility of the LHS 1,2,3-triazole in VHL-recruiting degraders has since been demonstrated in several publications and patents, including a 2024 patent by Astellas Pharma, Inc. (Astellas) likely covering their clinical degrader **ASP3082**. The patent shows that their LHS triazole compound (**64**, Figure 12) could effectively degrade KRAS G12D mutants and significantly inhibit tumor growth in a range of xenograft mouse models, either as a monotherapy or in combination with other pharmacological agents [68].

**Figure 13.**
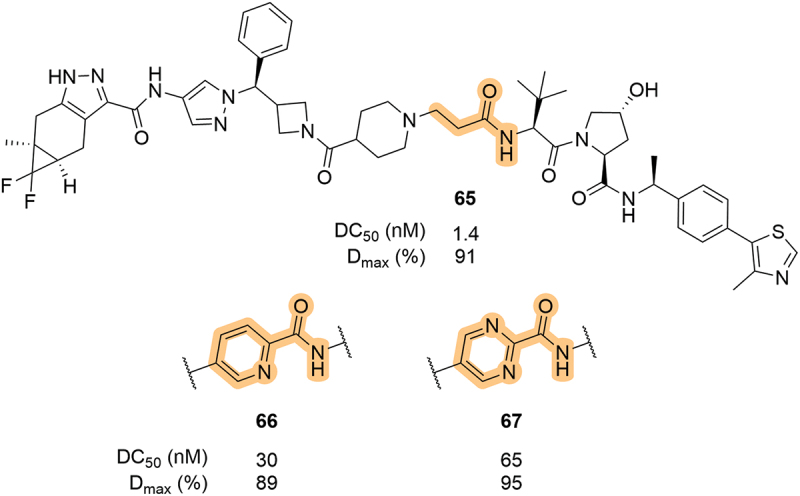
Pyridyl and pyrimidyl LHS amides utilised in ITK degraders patented by Nurix.

In 2023, Nurix Therapeutics, Inc. (Nurix) patented a series of VHL-based compounds as degraders of IL-2 inducible T-cell kinase (ITK) [69]. In this patent, few changes were made to either the ITK or VHL ligand and most of the optimization efforts were explored through linkerology. They did, however, report the use of pyridyl and pyrimidyl groups as part of the LHS amide as exemplified in **66** and **67** (Figure 13). Unfortunately, both **66** and **67** showed a significant rise (3- to 5-fold) in the DC_50_ value against ITK when compared to the linear alkyl counterpart, **65**. Interestingly, the D_max_ value was relatively unaffected.

**Figure 14.**
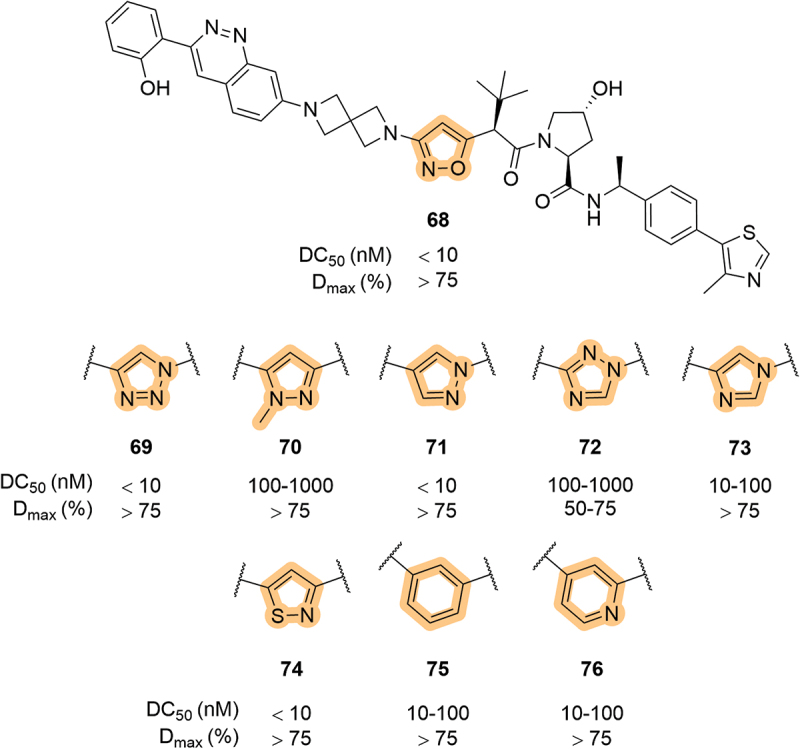
Five- and six-membered heterocycle mimetics of the LHS amide, used in SMARCA2/4 degraders patented by foghorn therapeutics. DC_50_ and D_max_ for SMARCA2.

Exploration of LHS amide mimetics was reported within a patent filed in 2023 by Foghorn Therapeutics [70]. Within the array of SMARCA2/4 degraders, there were many examples containing heterocycles in place of the LHS amide, a selection of LHS-linked examples is highlighted in Figure 14. As previously shown, LHS isoxazole-linked degraders can be highly effective as exemplified by **68**. Various five-membered nitrogen-containing heterocycles were patented, including the 1,2,4-triazole **72** which was significantly less potent (DC_50_ >10-fold higher) than the corresponding 1,2,3-triazole **69**. A notable finding was that of the LHS heterocyclic compounds, only the 1,2,3-triazole **69** showed significant degradation of SMARCA2, and the isothiazole **74** showed moderate SMARCA2 activity. This shows that these single-atom changes can manifest sizable changes in a compound’s biological activity. Interestingly, a phenyl and a pyridyl example (**75** and **76**, respectively) also showed substantial SMARCA2 degradation (DC_50_ 10–100 nM) despite these groups not being typical amide mimetics.

**Figure 15.**
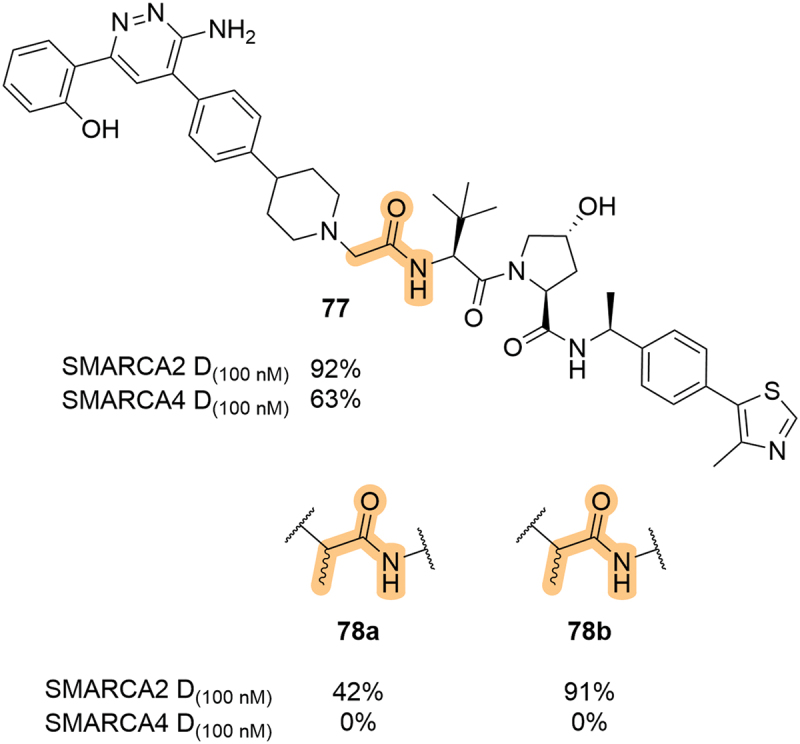
Incorporation of α-methyl groups on the LHS amide of SMARCA2/4 degraders, patented in 2023 by Aurigene. D_(100 nM)_ = degradation achieved after compound treatment at 100 nM.

Building on from an earlier patent published in 2019, Aurigene Discovery Technologies Ltd. (Aurigene) published a series of dual SMARCA2/4 degraders in 2023 [71,72]. Aiming to develop selective degraders of SMARCA2, the parent compound **77** showed significant degradation of both SMARCA2 and SMARCA4 at 100 nM (Figure 15). Interestingly, the introduction of a chiral methyl group at the LHS alpha position perturbed degradation of SMARCA4 whilst maintaining SMARCA2 activity. One isomer (**78b**) was significantly more potent than the other (**78a**), but unfortunately the absolute stereochemistry of the isomers was not determined and so the structural basis for this gained selectivity is difficult to rationalize. This use of a chiral methyl group alpha to the LHS amide was also covered in a patent by Treeline Biosciences (Treeline), although the two isomers displayed similar activity against their protein target, B-cell lymphoma extra-large (BCL-xL) [73].

**Figure 16.**
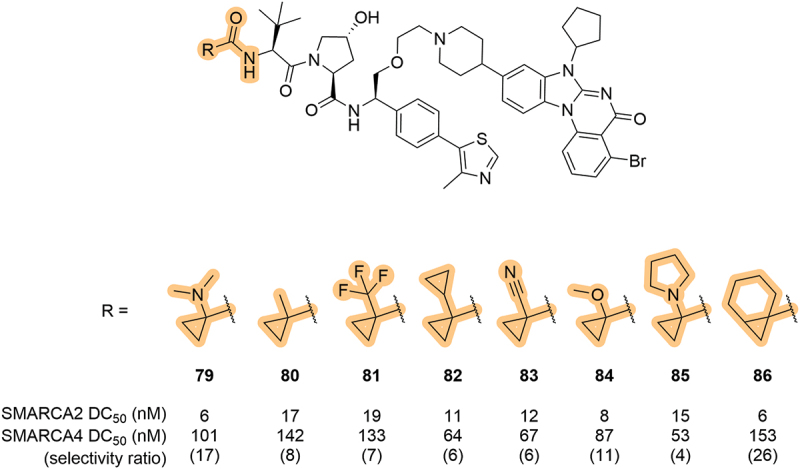
Systematic exploration of the cyclopropyl group in the LHS amide of SMARCA degraders patented by the Boehringer Ingelheim-Dundee collaboration in 2023.

In 2023, following on from an earlier 2020 patent, Boehringer Ingelheim International GmbH (Boehringer Ingelheim) and the University of Dundee patented a series of SMARCA2/4 degraders which resulted in the discovery of SMARCA2-selective degraders, including the *in vivo* orally efficacious compound **ACBI2** [74–76]. Within this patent, the terminal cyclopropyl moiety of the LHS amide was investigated with various substituents (Figure 16). SMARCA2 and 4 degradation was relatively insensitive to changes in this region of the VHL ligand, as exemplified by the relatively similar profiles of **79** and **86** despite differences in steric bulk and polarity. The tolerance of heteroatoms in this region of the VHL ligand likely led to benefits to the physicochemical properties of the compounds without compromising on the potency or selectivity.

**Figure 17.**
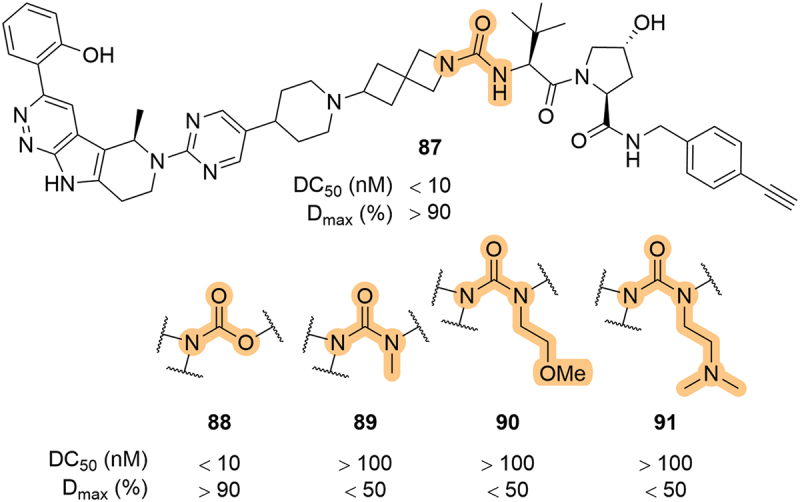
SMARCA2/4 degraders patented by Kymera in 2023 bearing LHS urea and carbamate linkages.

Building on from several earlier patents, Kymera published a series of SMARCA2/4 degraders that contained a urea linkage between the linker and LHS of the VHL ligand (Figure 17) [57,59,60,63,77,78]. The spiro[3.3]heptane urea was conserved among many of the patented compounds, although systematic modification of the urea moiety was reported. Carbamate **88** showed similar potency to the parent urea (**87**), however, substitution of the urea with a methyl group (**89**) or alkyl chains bearing solubilizing groups (**90** and **91**) had a detrimental effect on potency. It is also worth noting that in an earlier patent, Kymera showed that the incorporation of a LHS urea moiety was detrimental for activity against both SMARCA2 and 4, as was found by Arvinas in their BRAF degraders [52,57]. Kymera further reported BCL-xL degraders that were linked via a LHS urea, although in that case the azetidyl-urea remained equipotent to the corresponding amide [79].

Despite the developments in other positions since 2019, the LHS amide remains one of the most common linker attachment points in the VHL ligand today. The wide tolerance of linkers and synthetic ease at which they can be installed, still make this exit vector an attractive choice for bespoke SAR but also for early automated chemistry library synthesis for ‘direct-to-biology’ (D2B) approaches. Since 2019, there has been a significant rise in the use of heterocycles as LHS amide mimetics, with five-membered heteroaromatic groups such as isoxazole and 1,2,3-triazole becoming the new benchmarks, particular among patented degraders with potential clinical scope. When LHS amides are still used in contemporary literature, aromatic and saturated heterocycles are often used, blurring the line between VHL ligand and linker.

## 3. *Tert*-leucine modifications

The *tert*-leucine region of the canonical VHL ligand scaffold was crucial to breaking the ‘nanomolar’ affinity threshold early on in compound optimization. While peptidic in nature, prior to 2019, *tert*-leucine had seen little variation in the context of VHL-based degraders other than being replaced with alanine, threonine, valine or penicillamine, the latter of which provided a convenient handle for linker attachment [19,22,23,31]. Despite initially being kept unchanged, the *tert-*leucine side chain points out from the groove of the VHL binding site (Figure 3(b)) and thus is a convenient handle for modulation and expansion.

**Figure 18.**
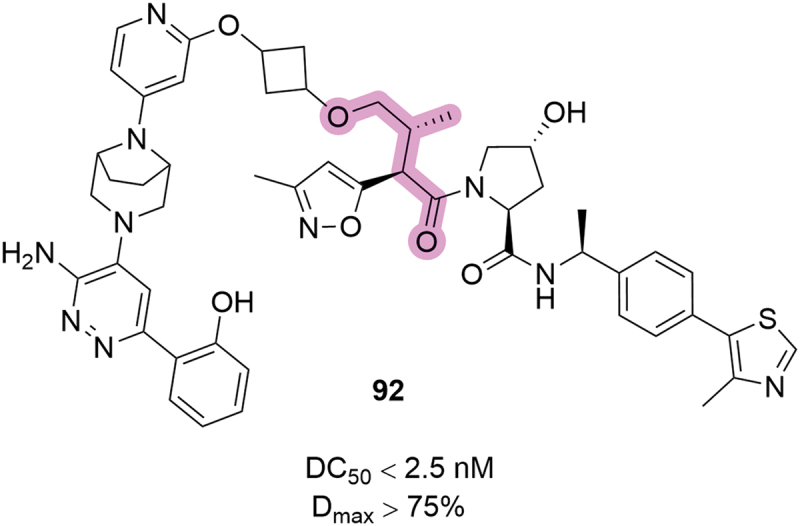
VHL-recruiting SMARCA2/4 degrader **92** patented by Arvinas with an ether tether from the linker to the valine part of the VHL ligand.

In 2019, Arvinas patented a series of SMARCA2/4 degraders that covered a range of different POI ligands, linkers, and E3 ligases that were recruited [48]. Among the VHL-based examples, **92** was reported, where the isopropyl group of the valine derivative was modified to include a pendant ether motif that the POI ligand and linker could be conjugated to (Figure 18). This ether compound was a potent degrader of both SMARCA2 and 4 in cellular assays. Linkage to the VHL ligand through this position has been extensively reported in other recent patent literature [57,59,80–83].

**Figure 19.**
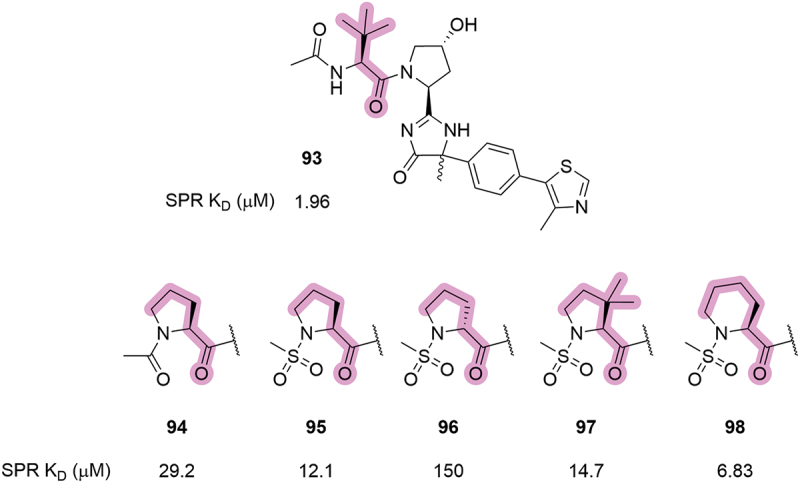
Cyclisation of the *tert*-leucine side chain in VHL inhibitors patented by Genentech in 2019. Corresponding K_D_ values determined by SPR.

A series of interesting modifications to the LHS of the VHL ligand were patented by Genentech in 2019 [53]. In this patent, some of the disclosed compounds contained a pyrrolidine or piperidine which was capped with either an acetyl or methanesulfonyl group (Figure 19). This cyclization from the canonical *tert*-leucine group on to the LHS amide led to some loss in binding affinity for VCB compared to the parent compound **93** (a 10-fold increase in the K_D_ values, as measured by SPR). Compound **96** showed a further 10-fold drop in binding affinity relative to its epimer, **95**, which highlighted that the stereochemistry of the alpha position was still important to consider even when the side chain is cyclized. Addition of a geminal dimethyl group (**97**) showed comparable affinity as the parent compound (**95**) and increasing the size of the ring by an additional methylene unit (**98**) gave a 2-fold improvement in affinity. Overall, cyclization at this position was unfavorable for VHL binding affinity and has also been shown to be detrimental for degradation potency of AR degraders (>40-fold increase in DC_50_) in a 2023 patent by Suzhou Kintor Pharmaceuticals, Inc. (Suzhou Kintor) [84].

**Figure 20.**
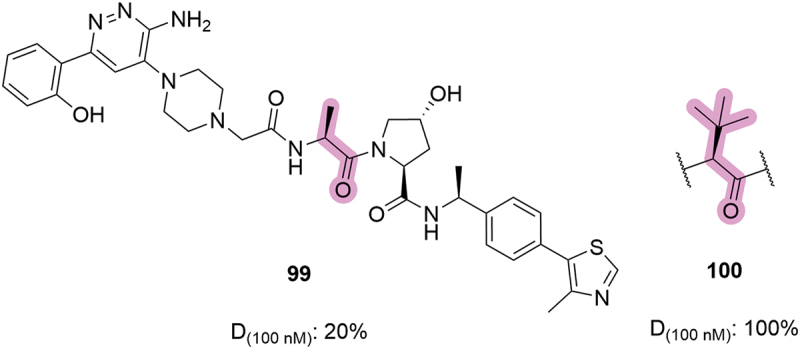
Truncation of the *tert*-leucine side chain in SMARCA2/4 degraders patented by Aurigene in 2019. D_(100 nM)_ = degradation achieved after compound treatment at 100 nM.

Early patent literature by Aurigene also showed that truncation of the *tert*-butyl side chain was detrimental for degradation potency (Figure 20) [71]. This is consistent with the loss of binary VHL binding affinity from replacing the *tert*-butyl with a methyl side chain in the context of **VH032**, as reported by Soares *et al*. [23]. Compound **99**, which only contained a methyl side chain, reduced SMARCA2 degradation levels by 80% points when compared to the analogous *tert-*leucine compound **100**.

**Figure 21.**
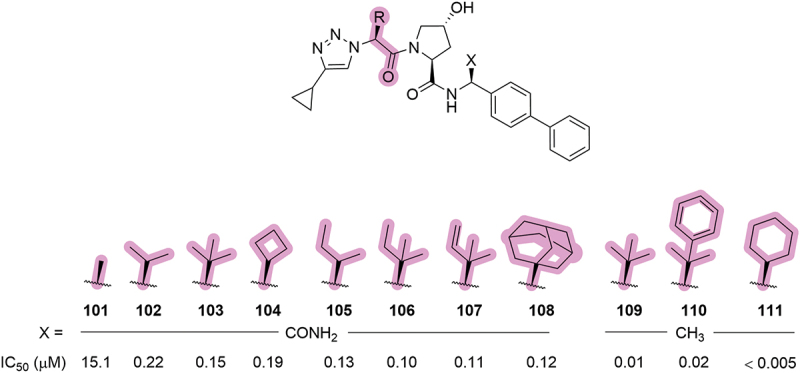
Modulation of the *tert*-leucine side chain in VHL inhibitors patented by Genentech in 2022. corresponding IC_50_ values were determined by a NanoBRET cellular assay.

In addition to modification of the LHS triazole substituent (see section 2), a 2022 patent by Genentech replaced the *tert*-leucine side chain with lipophilic groups of varying size (Figure 21) [65]. As previously exemplified in **99** by Aurigene, truncation of the *tert*-butyl side chain to a methyl (**101**) was detrimental to activity (100-fold increase in IC_50_ relative to **103**). It was also found that increasing the steric bulk of the side chain had little impact on the activity against VHL (e.g. **103** and **108** having similar IC_50_ values) and that the cyclohexyl ring was the optimal side chain with **111** having an IC_50_ value below the limit of the assay (IC_50_ <0.005 µM).

**Figure 22.**
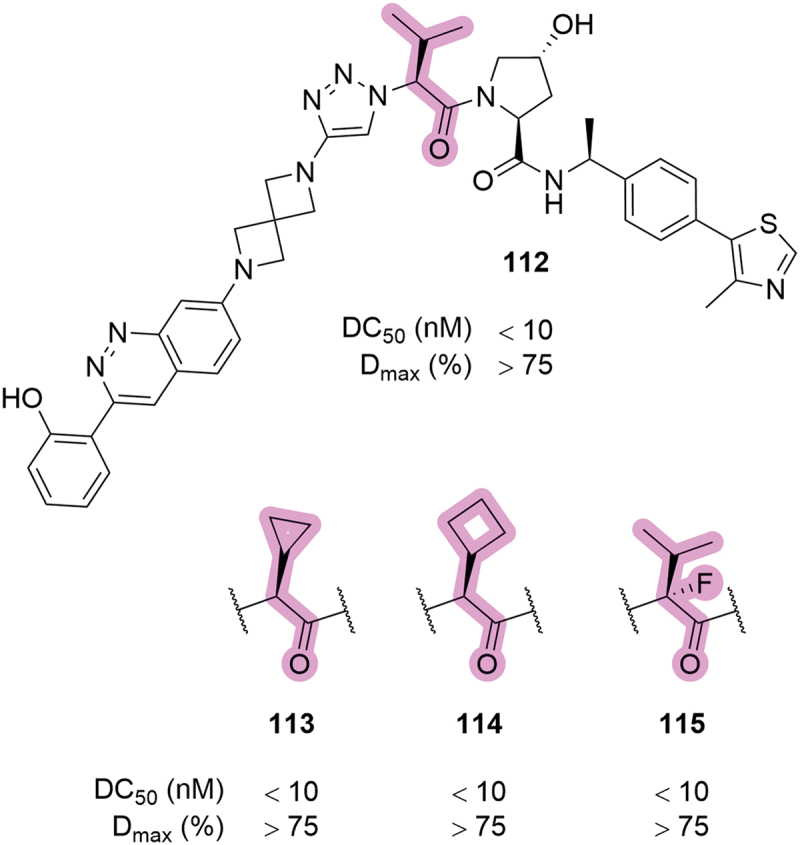
Modulation of the valine side chain in SMARCA degraders patented by Foghorn therapeutics in 2023, and the corresponding SMARCA2 degradation data.

In 2023, Foghorn Therapeutics patented a series of SMARCA2/4 degraders which contained modifications across many areas of the compound’s structure, including the *tert*-leucine group (Figure 22) [70]. The side chain was replaced with isopropyl (**112**), cyclopropyl (**113**) and cyclobutyl (**114**) groups – all of which showed similar potent degradation of SMARCA2 in HiBit knock-in HeLa cells (DC_50_ <10 nM). In this patent, Foghorn Therapeutics also disclosed compound **115**, which contained a fluorine atom at the alpha position of the valine moiety and showed comparable degradation potency to the parent compound **112**. Alpha fluorination of the *tert*-leucine group was also claimed by Arvinas in an earlier patent, although they did not report any biological data for the associated compound [52]. It is likely that the introduction of a fluorine atom at this position was intended to improve the pharmacokinetic properties or to prevent racemization of the stereocentre, although no data has been published to assess this.

**Figure 23.**
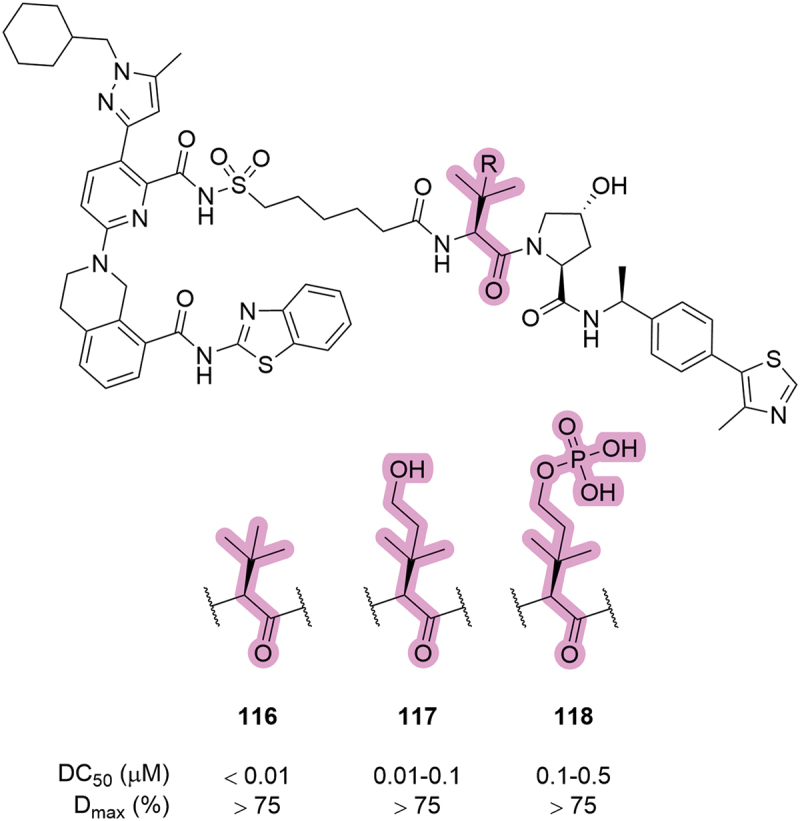
Modulation of the *tert*-leucine side chain in BCL-xL degraders patented by Kymera in 2023, and the corresponding degradation data.

In 2023, Kymera patented a series of compounds that recruited VHL for BCL-xL degradation [79]. Within the array of disclosed compounds, some unique modifications were made to the side chain of the *tert*-leucine moiety (Figure 23). The addition of polar groups, such as hydroxymethyl (**117**) and methylphosphate (**118**) were likely incorporated in order to improve aqueous solubility, although they reduced the effectiveness of cellular BCL-xL degradation. This loss of cellular activity was more pronounced with the phosphate analog (**118**) which is liable to poor cell permeability due to its anionic nature under physiological conditions.

**Figure 24.**
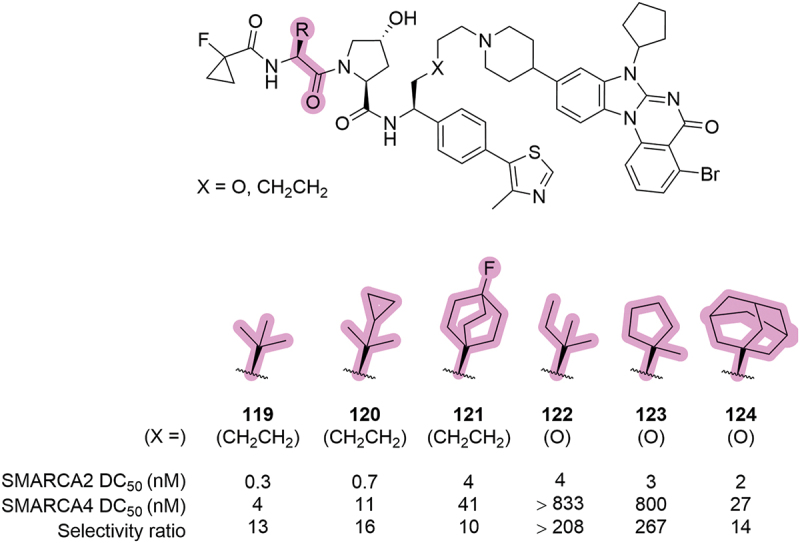
Modulation of the *tert*-leucine side chain in SMARCA2/4 degraders patented by Boehringer Ingelheim and the University of Dundee in 2023, and their corresponding DC_50_ values.

Within a series of SMARCA2/4 degraders patented by Boehringer Ingelheim and the University of Dundee in 2023, there was a selection of compounds which had unique changes to the *tert*-leucine side chain [76]. Within the range of reported modifications, there was the addition of lipophilic substituents to the *tert*-butyl group, such as cyclopropyl **120** and methyl **122**, and bulky cyclic variants such as fluoro-bicyclo[2.2.2]octane **121**, methylcyclopentyl **123** and adamantyl **124** (Figure 24). All of the reported modifications led to reduced degradation of SMARCA2 and SMARCA4, which resulted in the *tert*-leucine group remaining unchanged in the lead compound, **ACBI2** (**119**) [75].

**Figure 25.**
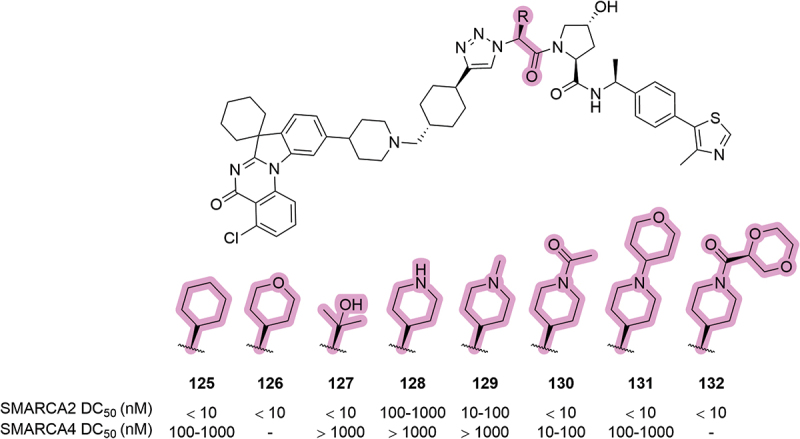
Heterocyclic modifications of the *tert*-leucine side chain in SMARCA degraders patented by the University of Michigan in 2024 and the corresponding degradation data. ‘-’ indicates data not reported.

In a 2024 patent by the University of Michigan, a series of SMARCA2/4 degraders were disclosed, some of which had modifications to the *tert*-leucine side chain (Figure 25) [85]. The side-chain modifications reported within this patent were primarily centered around six-membered heterocycles such as pyran **126** and *N*-substituted piperidines **128-132**. Most of the reported modifications showed comparable SMARCA2 activity as the parent cyclohexyl compound **125**, except for the *N*-Me and *N*-H compounds **129** and **128**, which showed roughly a 10-fold and 100-fold increase in DC_50_ values, respectively. The two examples containing larger side chains, **131** and **132**, showed good retention of SMARCA2 activity which suggests a good tolerance for expansion as this position of the VHL ligand – which further validates this position as an attractive attachment point for linkers. The same side-chain modifications were also tolerated in another patent by the University of Michigan, which disclosed a series of VHL-based degraders of Signal transducer and activator of transcription 3 (STAT3) [82].

Since 2019, modifications to the *tert*-leucine group have focused primarily on the side chain, with the main efforts focused on increasing steric bulk to try and improve binding affinity and/or the incorporation of heteroatoms and polar groups to try and reduce lipophilicity. Overall, efforts to improve affinity have generally been met with low success and as a result the canonical *tert*-butyl group remains dominant in reported compounds. It is unclear whether the introduction of heterocycles at this position led to any meaningful changes to the physicochemical properties of the compounds as this information is rarely reported in patent literature.

## 4. RHS amide modifications

In this review, the RHS amide region of the VHL ligand includes the amide in the 2-position of the Hyp core and the contiguous benzylic position that connects the mentioned amide with the phenyl ring of the ligand (Figure 4). The RHS amide forms important interactions with the VHL’s protein surface that anchors the RHS of the ligand, but also carries a hydrogen-bond donor and high desolvation energy penalty that can be detrimental for physicochemical properties of the PROTACs. Due to the physiochemical drawbacks of the amide, several examples have been patented trying to replace or modify this region of the ligand. Furthermore, the benzylic position was initially substituted with an (*S*)-Me to improve affinity to VHL and has extensively been used as an exit vector for PROTACs, demonstrating that there is space for modification/substitution [26].

**Figure 26.**
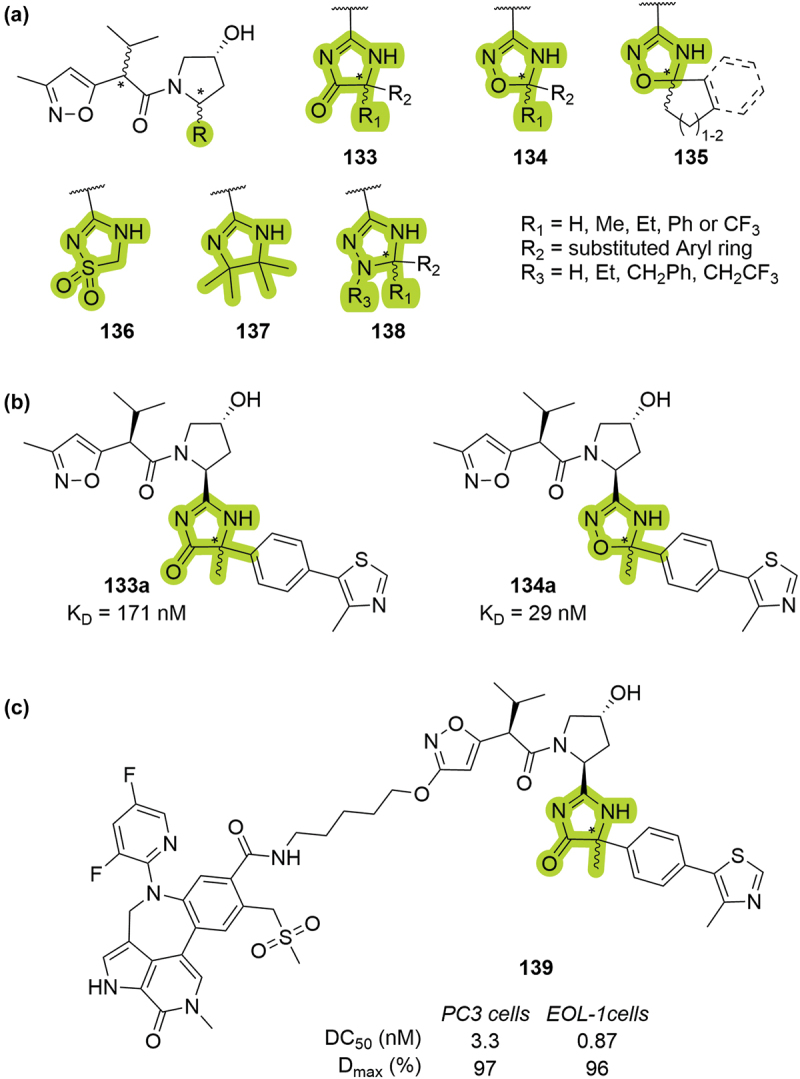
Early attempts of bioisosteric replacement of the RHS amide, patented by Genentech. (a) VHL ligands with heteroaryl groups in place of the RHS amide. (b) Examples of VHL ligands with better affinity for VHL; K_D_ reported from SPR assay. (c) BRD4 targeting PROTAC **139**.

In 2019, Genentech patented two series of degrons and PROTACs for targeted protein degradation of BRD4. In the first patent, the VHL-based degrons featured different heterocyclic groups diversly substituted as isosteres of the RHS amide (Figure 26(a)) [53]. The affinity of these VHL ligands was investigated by SPR, returning a wide range of K_D_, from high µM to low nM values. The best binding affinities were obtained for one of the two possible diastereomers of compounds **133a** and **134a**, showing K_D_ of 171 nM and 29 nM respectively (Figure 26(b)). In addition, it was demonstrated that these heterocyclic VHL ligands were well-tolerated in PROTACs, exemplified with compound **139** (Figure 26(c)), which proved to be very potent in PC3 and EOL-1 cell lines (DC_50_ = 3.3 nM and 0.87 nM and D_max_ = 97% and 96% respectively).

**Figure 27.**
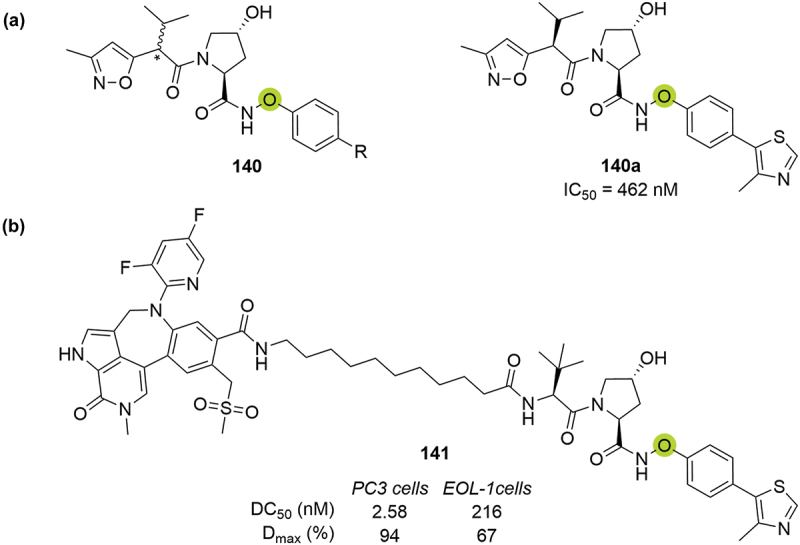
*N*-oxo-amide modifications patented by Genentech in 2019. (a) Examples of *N*-oxo-amide VHL ligands **140**. (b) BRD4 targeting PROTAC **141**.

In the second patent, Genentech disclosed different VHL ligands bearing an *N*-oxo-amide in place of the RHS amide (**140**), displaying different affinities for VCB with a range of K_D_ values from high µM to low nM (Figure 27(a)) [86]. As an example, compound **140a** showed VHL IC_50_ of 462 nM measured by FP. Representative PROTAC **141** resulted in a more potent degrader of BRD4 in PC3 than EOL-1 cell lines (DC_50_ = 2.58 nM and 216 nM and D_max_ = 94% and 67% respectively).

**Figure 28.**
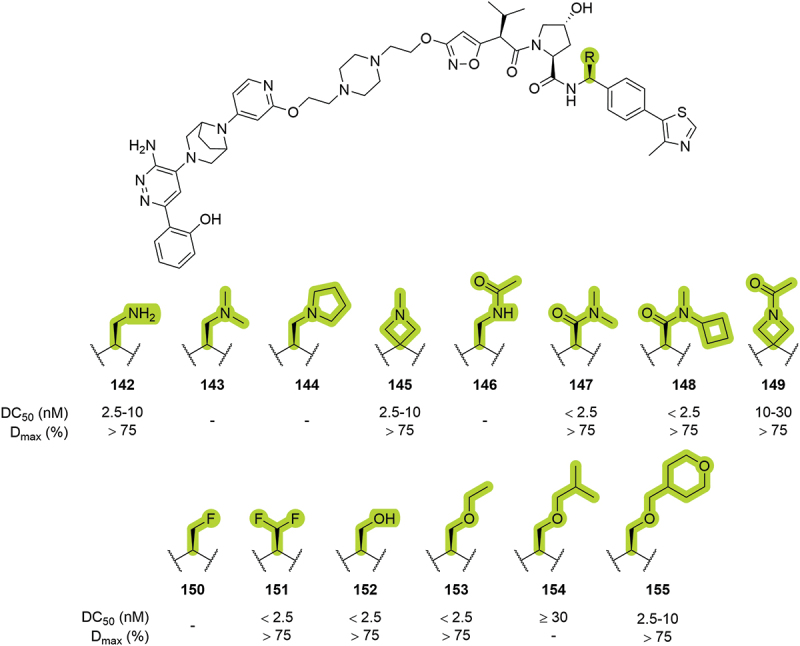
Benzylic substitutions patented by Arvinas in 2019. ‘-’ indicates data not reported.

In the same year, Arvinas disclosed a series of dual degraders targeting SMARCA2/4 [48]. Among other modifications, the authors extensively substituted the benzylic position of the VHL ligand with primary and tertiary amines (**142-145**), secondary and tertiary amides (**146-149**), fluorine atoms (**150**, **151**), hydroxyl group (**152**) and ethers (**153–155**). All the PROTACs with a common structure bearing these modifications showed a maximal degradation of SMARCA2 superior to 75%, and DC_50_ lower than 30 nM, except for isobutyl ether substituted **154** (Figure 28). A posterior patent published in 2021 by Janssen Pharmaceutica NV (Janssen) showed the utilization of the fluoro-methylene (as in compound **150**) as a benzylic substitution in the context of Interleukin-1 receptor-associated kinase 1 (IRAK1) degradation [87]. In 2023, Astellas disclosed different PROTACs targeting KRAS^G12D^ bearing the same benzylic substitutions as in **143** and **150-152**. Bifunctional degraders with a hydroxymethyl group in the benzylic position (as in **152**) showed tumor growth inhibition rates between 74% and 90% in human KRAS^G12D^ mutation positive GP2d colorectal cancer cell line [88].

**Figure 29.**
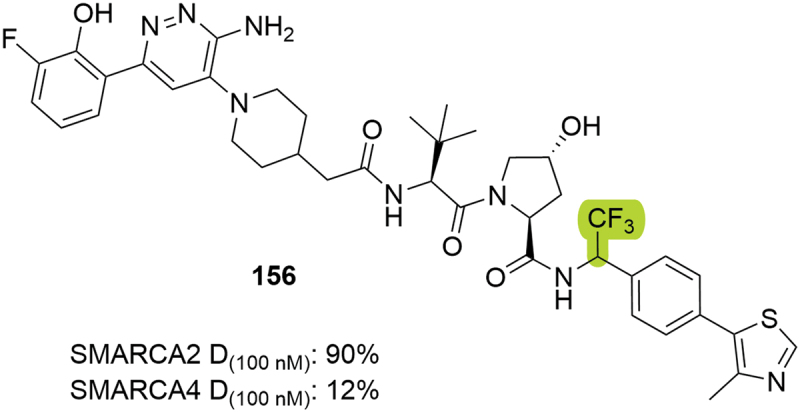
SMARCA2 degrader **156** patented by Aurigene. D_(100 nM)_ = degradation achieved after compound treatment at 100 nM.

In the context of SMARCA2/4 degradation, Aurigene patented bifunctional degrader **156**, where a trifluoromethyl group was introduced in the benzylic position (Figure 29) [71]. The racemic mixture in this position was tested in H929 cells resulting in selective degradation of SMARCA2, with 90% reduction at 100 nM, compared to 12% reduction of SMARCA4. The utilization of this substituent was posteriorly patented by Treeline in bifunctional degraders targeting BCL-xL [73].

**Figure 30.**
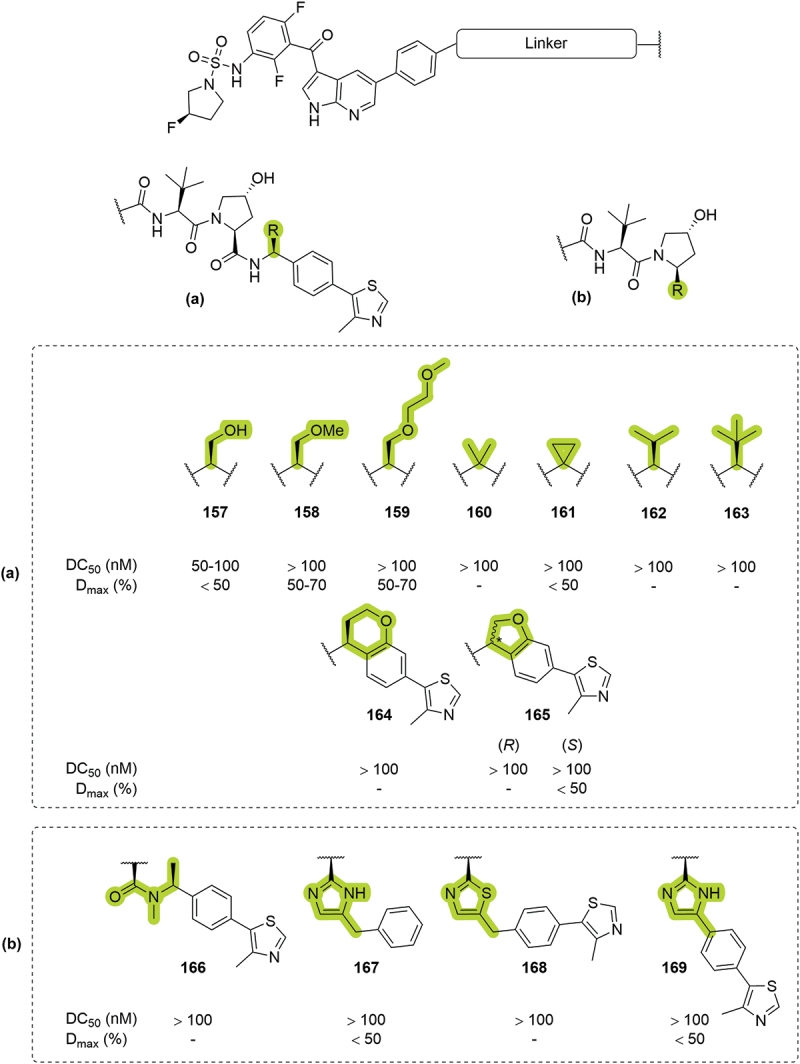
RHS amide modifications patented by Arvinas in 2020. (a) Substituents introduced in the benzylic position. (b) Amide modifications, including H-bond donor removal and amide isosteres. ‘-’ indicates data not reported.

In 2020, Arvinas patented a series of degraders targeting BRAF proteins [52]. In this disclosure, the authors introduced different hydroxyl/ether (**157-159**) and alkyl (**160-163**) substituents at the benzylic position of VHL-based PROTACs with poor degradation profiles (Figure 30(a)). Within the patented compounds, the use of cyclized ethers such as chromane **164** or benzofuranes **165** to rigidify the ligands returned DC_50_ >100 nM and D_max_ <50%. The methylation of the amide as an H-bond donor removal strategy (**166**) or the utilization of imidazoles (**167** or **169**) and thiazole (**168**) as amide isosteres also returned compounds with poor degradation profiles (Figure 30(b)). Further patents from Kymera included cyclopropyl as a benzylic modification for degradation of SMARCA2/4, and along with hydroxymethyl and methoxymethyl for degradation of BCL-xL, providing good degradation results for both proteins [57].

**Figure 31.**
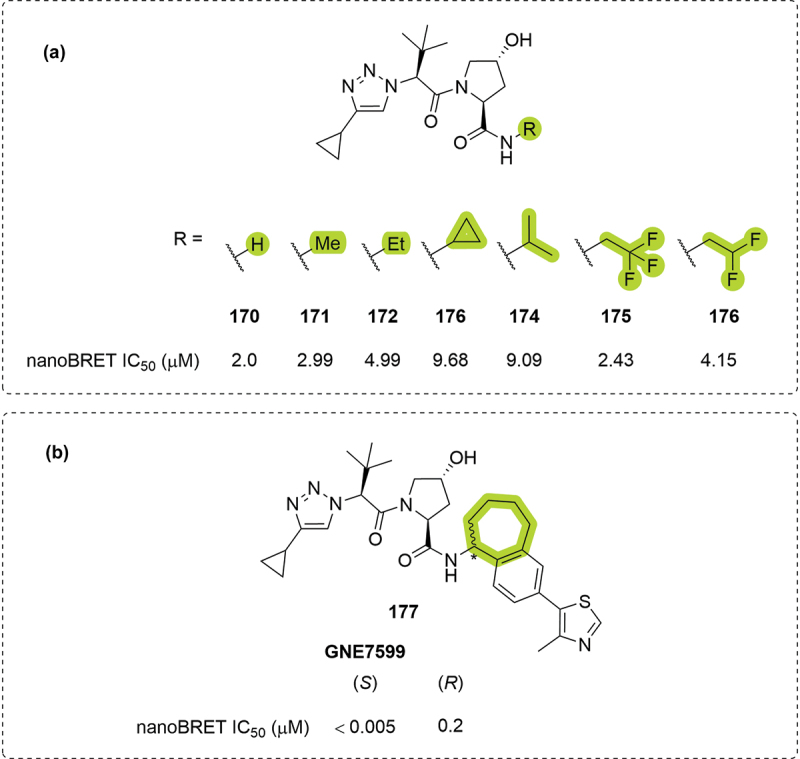
VHL ligands reported by Genentech in two different patents in 2022. (a) Truncated VHL ligands. (b) Cycloheptyl ring modification introduced in compounds **177**, resulting in the discovery of **GNE7599**, the most potent VHL ligand reported so far.

In 2022, Genentech reported two series of VHL inhibitors in two different patents that included modifications in the RHS benzylic region. In one patent, a series of truncated VHL ligands was reported (Figure 31(a)) [65]. Within the patented compounds, the RHS amide was left either unsubstituted (**170**) or substituted with alkyl chains (**171-174**) and fluoro-alkyl chains (**175**, **176**). These compounds did not show a good affinity for VHL, returning IC_50_ >2 μM in nanoBRET assays. This loss of affinity could be explained by the truncation of the RHS region, suggesting that these groups are ultimately not good surrogates for the contiguous phenyl-thiazole group. In the other patent, the optimized compound **177** was disclosed (Figure 31(b)), showing an IC_50_ <5 nM for the (*S*) diastereomer (**GNE7599**) [64]. This finding was subsequently published in 2024, and **GNE7599** is, to our knowledge, the most potent VHL ligand reported so far, with a K_D_ = 540 pM by SPR, and improved oral bioavailability (F = 46.5%) [89].

**Figure 32.**
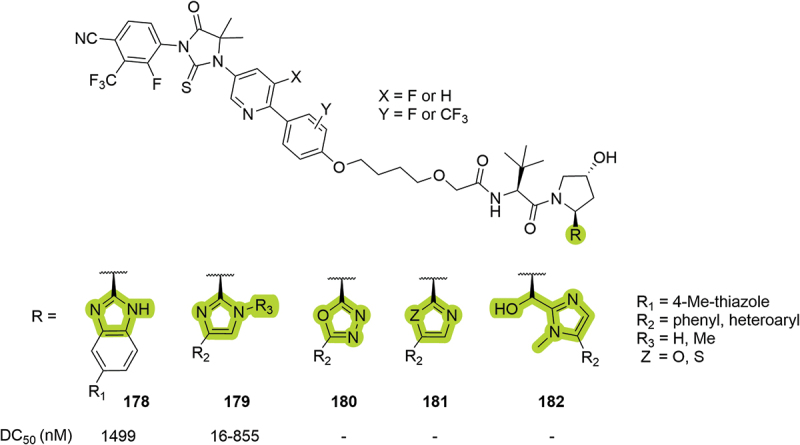
RHS amide isosteres patented by Suzhou Kintor in 2023 for the degradation of AR. Benzoimidazole **178**, imidazole **179**, oxadiazole **180**, oxazole and thiazole **181**, hydroxymethyl imidazole **182**. ‘-’ indicates data not reported.

The year after, Suzhou Kintor disclosed their work targeting AR. Among other modifications in the VHL binding moiety, the authors introduced different heteroaromatic groups as isosteres of the RHS amide (Figure 32). Bifunctional degraders bearing imidazole **179** returned the best degradation results (DC_50_ between 16 and 855 nM in LNCaP cells), and benzoimidazole derivative **178** was tested *in vivo* returning an eAUC_0.5-2 h_ of 471 ± 288 ng·h/mL after intragastric administration in mouse [84]. The same year, Mitsubishi Tanabe Pharma patented a series of VHL ligands bearing imidazole **179** (R_3_ = H) as an isostere of the RHS amide [90].

**Figure 33.**
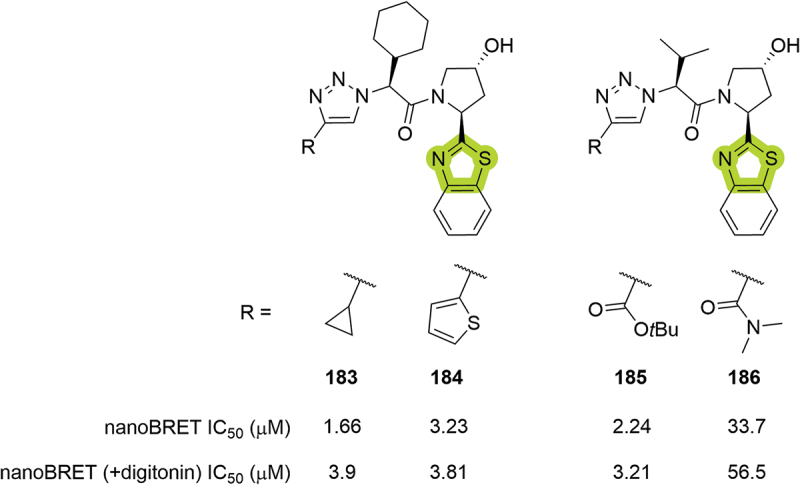
VHL inhibitors patented in 2023 by Genentech.

The same year, Genentech disclosed another series of VHL inhibitors bearing a benzothiazole as a RHS amide isostere (Figure 33). Although it is an interesting replacement, due to the removal of one H-bond donor and rigidification of the ligand, the patented compounds, which encompass different substituents in the LHS region of the ligands, did not show good inhibition of VHL in the nanoBRET assay returning IC_50_ between 1.66 and 33.7 μM [66].

**Figure 34.**
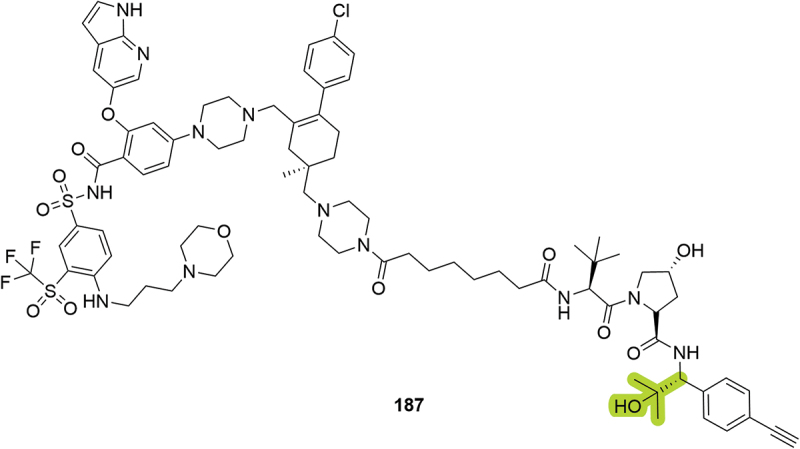
BCL-xL/BCL-2 PROTAC **187** patented by Kymera.

Also in 2023, Kymera Therapeutics patented their bifunctional degraders targeting BCL-xL/BCL-2 proteins [91]. Although no degradation data was reported, compound **187**, bearing a hindered hydroxyl in the benzylic position, was disclosed (Figure 34).

**Figure 35.**
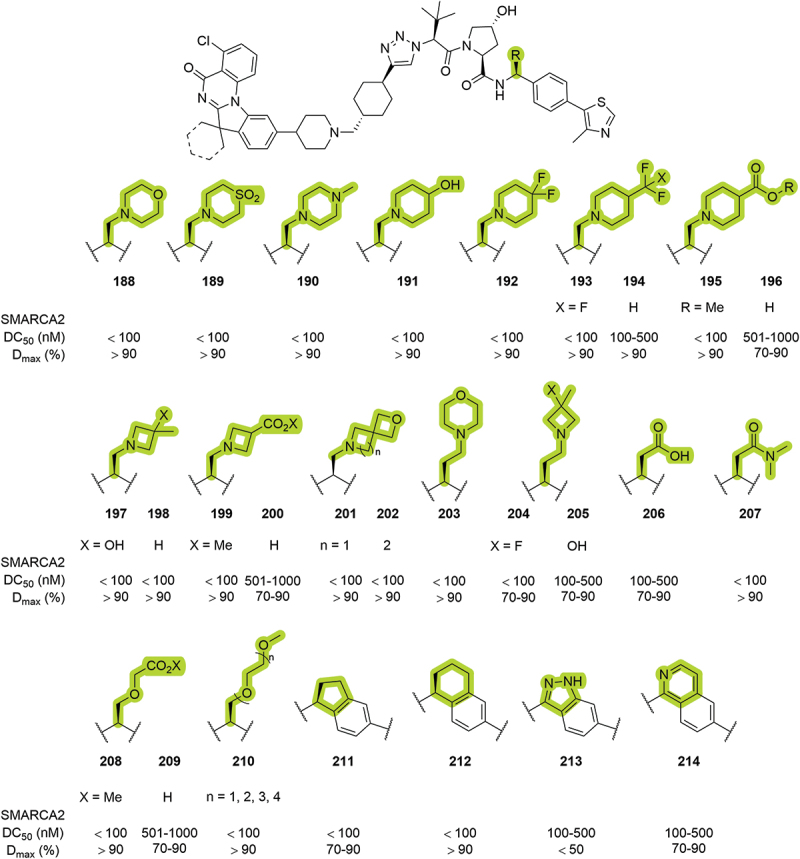
VHL-based PROTACs patented by the University of Michigan in 2024. SMARCA2 degradation reported from HiBiT assay.

In 2024, the University of Michigan patented a series of bifunctional degraders targeting SMARCA2/4 [85]. Within the patented compounds, a wide variety of substituents was introduced in the benzylic position (Figure 35). Different substituted cyclic amines, with one (**188-202**) or two (**203-205**) methylene groups as spacer, demonstrated good selectivity and excellent degradation of SMARCA2 in most of the cases, with DC_50_ <100 nM and D_max_ >90%. Carboxylic acids **196**, **206** and **209** were not well tolerated but the corresponding methyl esters **195** and **208** and dimethylamide **207** recovered degradation of SMARCA2. Methylated PEG chains **210** returned DC_50_ <100 nM and D_max_ >90%, in a similar way as the fused rings **211** and **212**, and the introduction of heteroaromatic rings, such as indazole **213** and isoquinoline **214**, decreased the degradation potential of the bifunctional degraders. In a later patent from the same group, morpholines such as **188** and **203**, and various length PEG chains, **210**, were utilized for targeted degradation of STAT3 [82].

Due to the peptidic nature of the VHL ligand, some strategies have been explored to introduce isosteric replacements of the RHS amide maintaining, in some cases, the affinity to VHL and resulting in good degraders. The contiguous benzylic position has seen more modifications in the last few years, bearing in mind its utilization as exit vector in the synthesis of PROTACs. Several substituents have been introduced, mostly as a strategy to rigidify the ligands or improve their solubility. These modifications can improve or maintain the degradation potential of the bifunctional degraders in a target-specific manner.

## 5. Phenyl group modifications

The phenyl ring of the VHL ligand has been extensively used as an exit vector in the synthesis of PROTACs, but its use as a handle for linkers in bifunctional degraders will not be discussed in the present review. The phenyl ring contributes to the affinity of the ligand to the VHL protein with aromatic interactions, which suggests its modification could improve such interactions. The phenyl region, neighbor of the RHS amide, could be used to shield the H-bond donor of the amide, and modifications toward this aim, which could improve the physicochemical properties of the compounds, have been explored.

**Figure 36.**
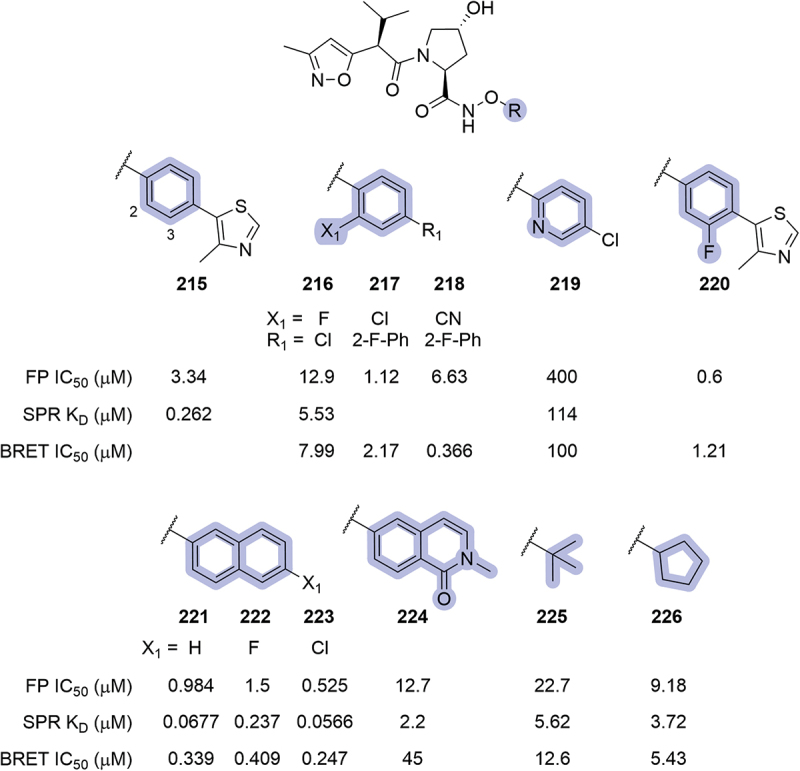
Phenyl modifications in VHL ligands bearing an *N*-oxo-amide in place of the RHS amide, as patented by Genentech in 2019.

In 2019, in the patent targeting BRD4 where Genentech disclosed different VHL ligands and PROTACs bearing an *N*-oxo-amide in place of the RHS amide, several modifications were introduced in the phenyl ring (Figure 36) [86]. Within the patented VHL ligands, substituents in the 2-position of the phenyl ring (**216-219**) were introduced without improving the affinity or inhibition of VHL. The introduction of a fluorine atom at the 3-position (**220**) returned a roughly 5-fold improvement in IC_50_ compared to the unsubstituted parent compound **215**. Interestingly, the replacement of the phenyl ring with naphthalene (**221**, or halogen substituted **222** and **223**) improved the inhibition potential of the ligands and returned sub-μM K_D_ by SPR. On the other hand, the replacement of the phenyl ring with isoquinolinone **224** or aliphatic substituents (**225**, **226**) was detrimental. These modifications, however, were not further applied in the synthesis of PROTACs targeting BRD4.

**Figure 37.**
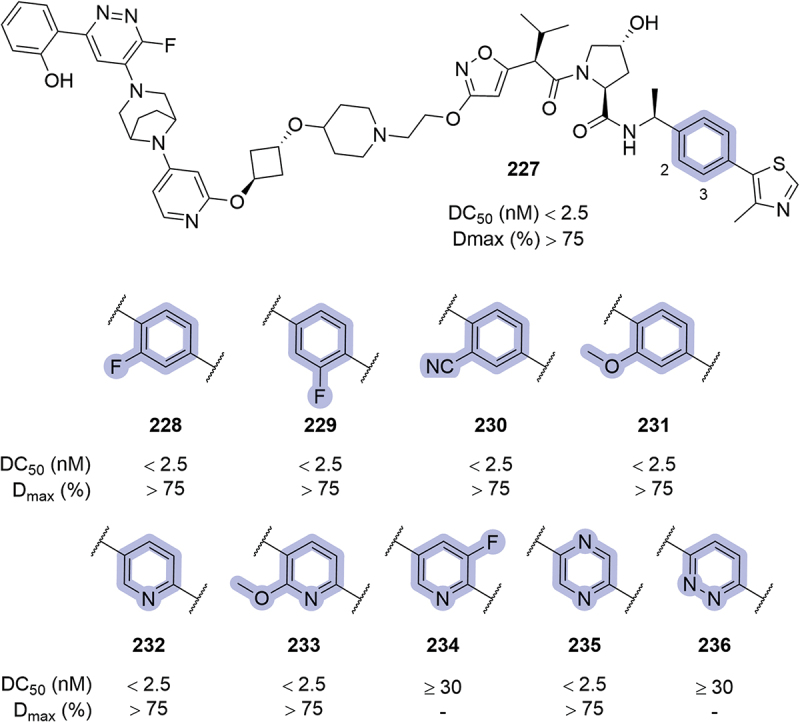
Phenyl modifications patented by Arvinas in 2019. ‘-’ indicates data not reported.

The introduction of fluorine atoms in the 2- or 3-position (**228** and **229**, respectively), or a nitrile group in the 2-position (**230**) was also utilized in bifunctional degraders targeting SMARCA2, patented by Arvinas the same year (Figure 37) [48]. Within the disclosed compounds, a 2-methoxy substituent was introduced (**231**), and the phenyl ring was replaced with heteroaromatic rings such as pyridines (**232-234**), pyrazine **235** or pyridazine **236**. All the modifications introduced resulted in very potent degraders, returning DC_50_ <2.5 nM and D_max_ >75%, except for compounds **234** and **236** where maximal degradation could not be calculated, with DC_50_ ≥30 nM. A posterior patent from Kymera in the context of targeted degradation of SMARCA2/4, pyridine and pyrimidine were also utilized as replacements of the phenyl ring, and Arvinas further explored the use of pyridine as phenyl replacement in 2023, in compounds targeting SMARCA2 [57,92].

**Figure 38.**
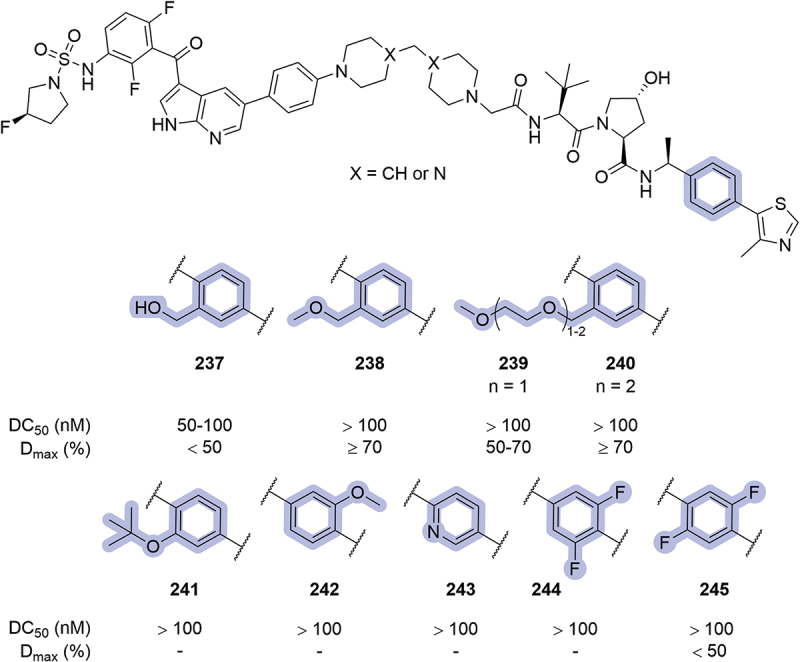
Phenyl modifications patented by Arvinas in 2020. ‘-’ indicates data not reported.

In 2020, in the patent where Arvinas disclosed their bifunctional degraders for BRAF proteins, modifications in the phenyl region were also reported (Figure 38) [52]. Substitution of the 2-position with hydroxymethylene (**237**) or methylene ethers (**238-240**) returned moderate degraders. The utilization of *tert*-butoxy (**241**) or methoxy (**242**) substituents in the 2- or 3-position respectively was not well tolerated, neither was 2-pyridine **243** or di-fluorinated phenyl rings (**244**, **245**). A posterior patent form Treeline utilized pyridine **243** as a replacement for the phenyl ring in bifunctional degraders targeting BCL-xL [73].

**Figure 39.**
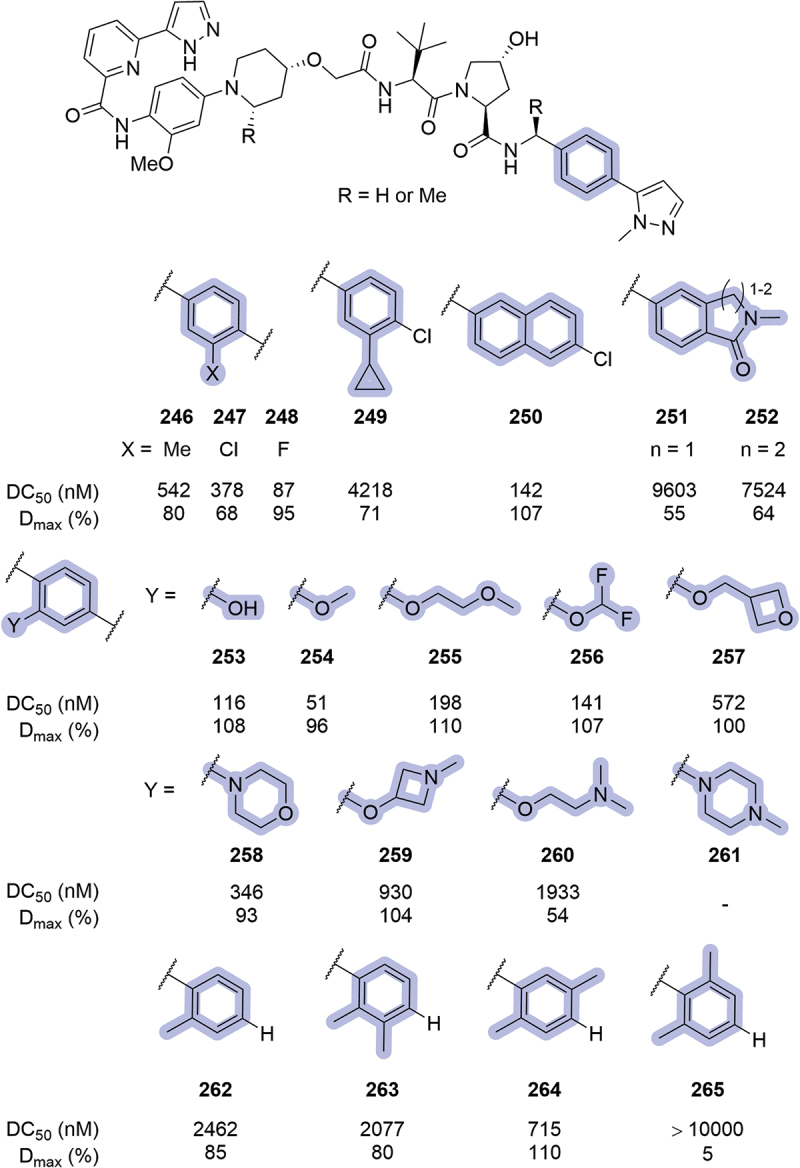
Phenyl modifications patented by Janssen in 2021. Degradation results from HiBiT assay with HEK293T cells. ‘-’ indicates data not reported.

In 2021, Janssen patented bifunctional degraders targeting IRAK1, introducing modifications in the phenyl ring region (Figure 39) [87]. The introduction of alkyl or chloro substituents at the 3-position of the ring (**246**, **247**, **249**) resulted in poor degraders, whilst the introduction of a fluorine atom in the same position (**248**) improved the maximal degradation up to 95%, with a DC_50_ of 87 nM. The replacement of the phenyl ring by Cl-naphthalene **250** resulted in a potent compound (DC_50_ = 142 nM, D_max_ = 107%), correlating with previously reported results by Genentech for this moiety (see Figure 36, compound **223**) [86]. Other bicycle replacements, such as lactams **251** and **252**, resulted in very poor degraders. A wide range of substituents was introduced in the 2-position of the phenyl ring such as hydroxyl **253**, ethers **254-257** and basic groups **258-261**. The most potent substituent was methoxy **254** (DC_50_ = 51 nM, D_max_ = 96%), while hydroxyl and ethers returned maximal degradation, and the introduction of basic groups was detrimental for degradation of IRAK1. Another set of modifications included the addition of methyl groups in the phenyl ring (**262-265**). These compounds lacked the terminal ring of the VHL ligand (as Me-pyrazole in this examples), and their degradation potency was reduced.

**Figure 40.**
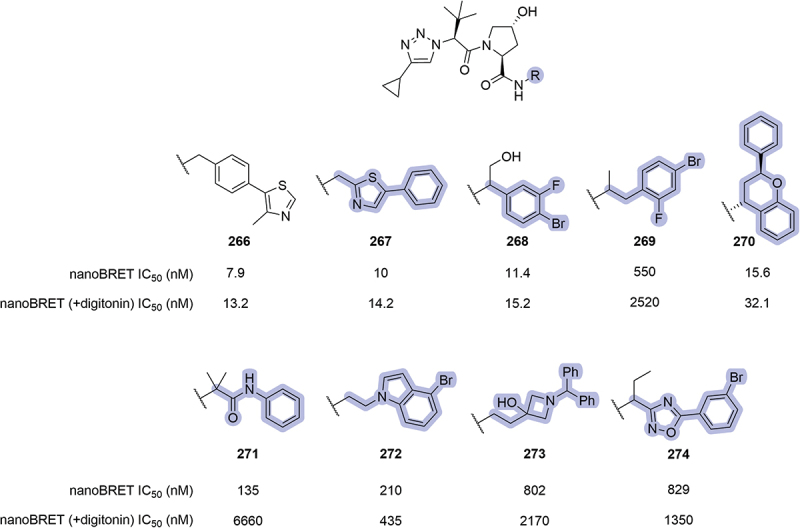
VHL ligands patented by Genentech in 2022 bearing phenyl ring modifications.

In 2022, Genentech disclosed a series of VHL inhibitors that encompassed a triazole ring in the LHS of the ligand [67]. Among the patented compounds, a wide range of modifications was introduced replacing the phenyl ring of the VHL ligand, the most potent ones illustrated in Figure 40. Compound **267**, where the position of the thiazole and the phenyl ring was exchanged, returned a similar inhibition of VHL to the parent compound (IC_50_ = 10 nM, compared to 7.9 nM for ligand **266**). The introduction of halogen atoms in the phenyl ring was tolerated, being compound **268** better than **269**. The inhibitory potential of the ligand was slightly reduced when the phenyl ring was replaced by chromane **270** (2-fold higher IC_50_ compared to **266**) and the introduction of amide **271** or indole **272** were tolerated. Interesting modifications such as the introduction of azetidine **273** and oxadiazole derivative **274** returned sub-μM IC_50_ values.

**Figure 41.**
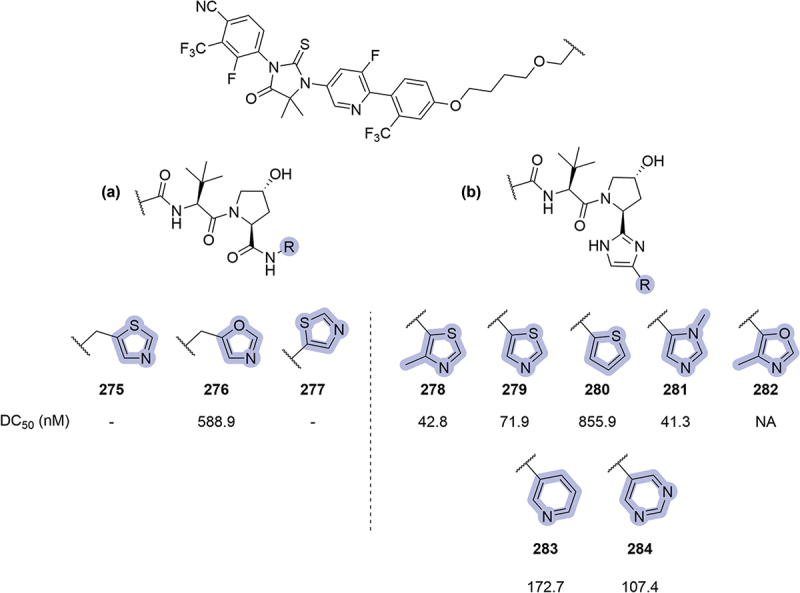
Phenyl ring replacements patented in 2023 by Suzhou Kintor. DC_50_ values obtained in LNCaP cells. ‘-’ indicates data not reported.

The year after, Suzhou Kintor disclosed their work targeting AR. In this patent, different replacements of the phenyl ring were reported in two different series of PROTACs, **A** and **B** (Figure 41) [84]. The introduction of five-membered heteroaromatic rings in series **A** did not result in potent degraders (**275-277**). When these rings were introduced in PROTACs from series **B**, bearing an imidazole as a RHS amide replacement, the resulting compounds (**278-282**) showed 2-digit nM DC_50_ in most cases. Pyridine **283** and pyrimidine **284** were introduced as well in this series, returning moderate DC_50_ values.

**Figure 42.**
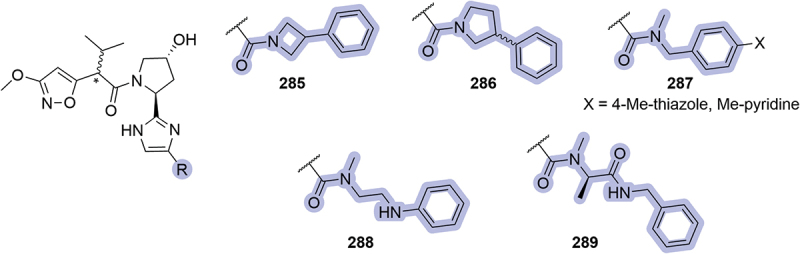
Modifications in the phenyl region reported by Mitsubishi Tanabe Pharma in 2023.

Also in 2023, Mitsubishi Tanabe Pharma patented a series of VHL ligands bearing an imidazole as an isostere of the RHS amide, introducing several modifications in the region of the phenyl ring [90]. Those modifications included different tertiary amides as a spacer between the imidazole isostere and the phenyl ring (Figure 42). Some representative examples, such as cyclic amides **285-286** and *N*-methyl-amides **287-289**, were investigated in SPR assays and displayed good affinity for VCB, with K_D_ <1 μM.

**Figure 43.**
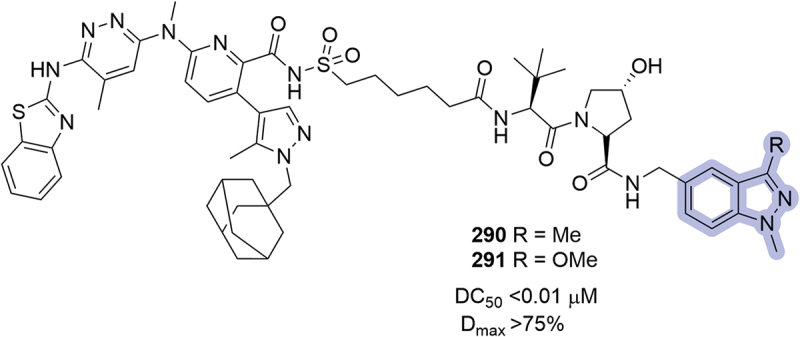
Indazole modifications introduced in PROTACs by Kymera in 2023. Degradation at 6 h in MOLT-4 in ELISA assay.

The same year, in the disclosure of BCL-xL degraders from Kymera, indazoles were introduced as phenyl ring replacements, as in compounds **290** and **291** (Figure 43). Both compounds returned DC_50_ <0.01 μM and D_max_ >75% [79]. The same modifications were introduced in a posterior patent by Kymera targeting BCL-xL/BCL-2 [91].

**Figure 44.**
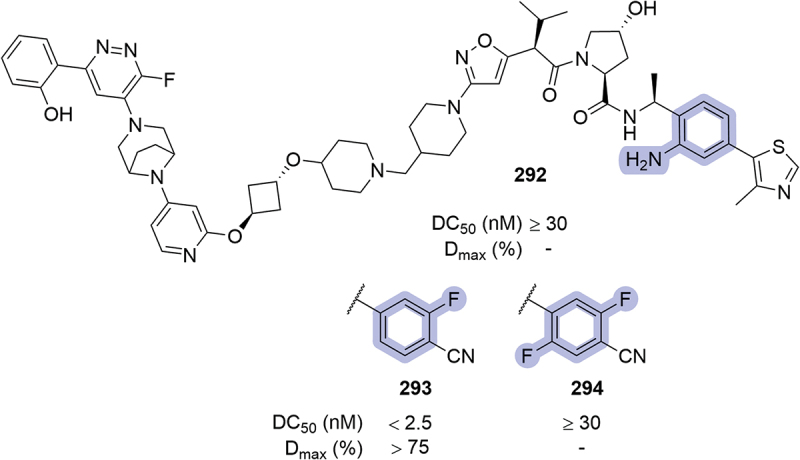
Phenyl substitutions reported by Arvinas in 2023. ‘-’ indicates data not reported.

In 2023, the disclosure by Arvinas targeting SMARCA2/4 proteins reported the use of an aniline to replace the phenyl ring, as in PROTAC **292** (Figure 44) [93]. This modification was not well tolerated, returning a DC_50_ ≥30 nM for SMARCA2 and D_max_ could not be calculated. The introduction of a fluorine atom at the 3-position, as previously reported, resulted in a very potent compound (**293**, DC_50_ <2.5 nM and D_max_ >75%), while the addition of a second fluorine atom *para* to the first (**294**) was detrimental (DC_50_ ≥30 nM).

**Figure 45.**
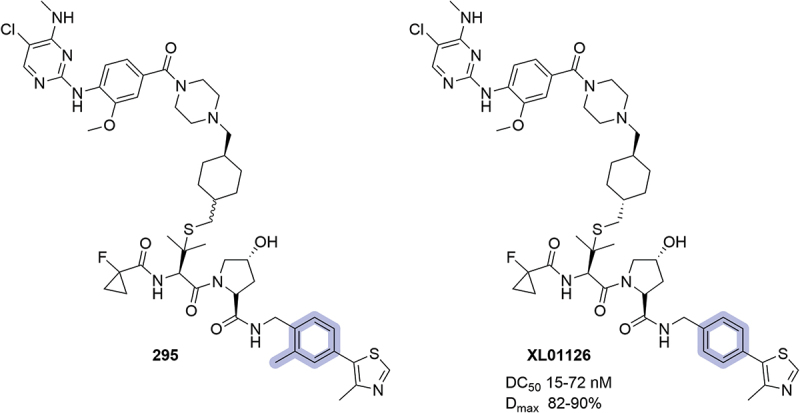
LRRK2 degraders patented by the University of Dundee in 2023. Degradation data for **XL01126** obtained in multiple cell lines.

Also in 2023, the University of Dundee disclosed a series of bifunctional degraders targeting leucine-rich repeat kinase 2 (LRRK2) [81]. Although the introduction of fluorine atoms at the 2- and 3-positions of the phenyl ring was also reported, it was the first time that the introduction of a methyl group at the 2-position was reported in the context of a ligand bearing the 4-methylthiazole as a terminal group (**295**, Figure 45). This modification, albeit it has been shown to maintain affinity for VCB, was not incorporated in the optimized oral and blood–brain barrier penetrant PROTAC **XL01126** published by the Ciulli lab [94,95].

**Figure 46.**
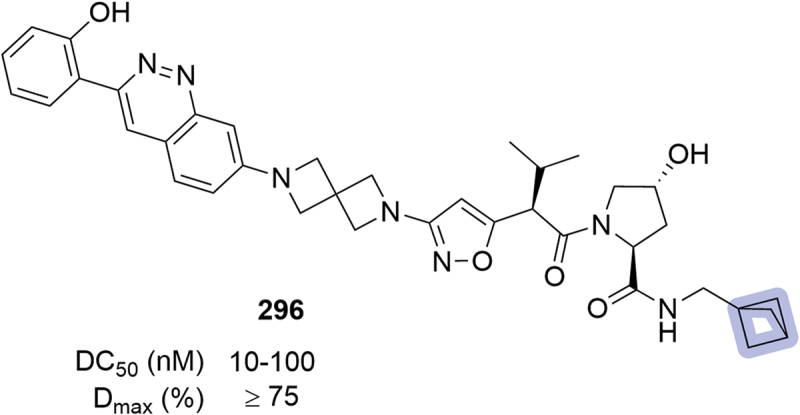
SMARCA2 targeting PROTAC **296** patented by foghorn therapeutics.

In the same year, Foghorn Therapeutics disclosed their work on bifunctional degraders targeting SMARCA2/4 for Brahma-associated factor (BAF) complex-related disorders. Among other modifications already commented in this section, compound **296** bearing bicyclo[1.1.1]pentane as bioisostere of the phenyl ring was reported, showing a DC_50_ between 10 and 100 nM and D_max_ ≥75% (Figure 46) [70].

**Figure 47.**
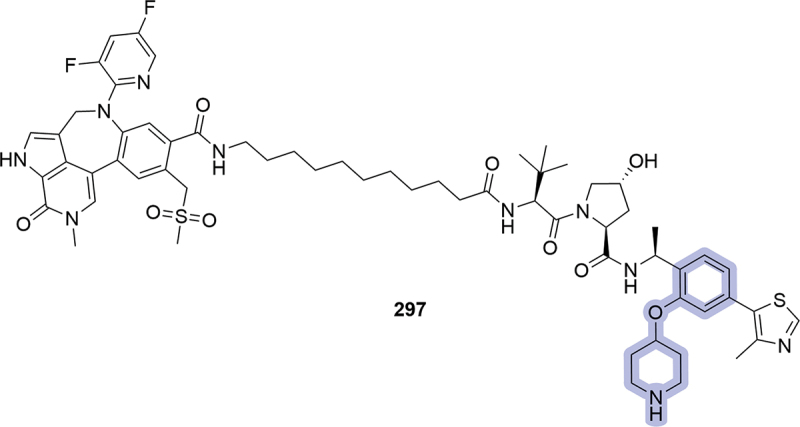
PROTAC **297** patented by Oerth Bio LLC in 2024 in the context of degradation in insects and insect cells.

In 2024, Oerth Bio LLC patented a series of bifunctional degraders targeting BRD3 and will-die-slowly protein (WDS) in insects and insect cells. An interesting modification in the phenyl ring region was the introduction of an oxygen-tethered piperidine in position two (**297**), although no degradation data was reported for this compound (Figure 47) [96].

The phenyl moiety of the VHL ligand has been generally maintained in the disclosed work presented here. The prevailing modifications in this region entail the introduction of different natured substituents in all the positions of the ring, from halogen atoms and basic carbocycles, up to aliphatic substituents or ethers, whilst the replacement of the ring by heteroaromatic or aliphatic rings have been also exploited. Whether it is to enhance the physicochemical properties of the compounds or their degradation potential, the phenyl ring of the VHL is drawing great attention from the TPD field.

## 6. 5-(4-Methyl)-thiazolyl group modifications

The 5-(4-Methyl)-Thiazolyl moiety of **VH032** and **VH101** (Figure 1) has become an established group within both VHL inhibitors and PROTACs, having been identified since early SAR as optimal at this position for high VHL binding affinity [20,22]. Expanding the scope of this region remains of great interest to improve various molecule parameters, including ‘drug-like’ properties and enhancing ternary complex formation in a PROTAC context. To further broaden the scope of the thiazole moiety many patents have disclosed numerous modifications.

**Figure 48.**
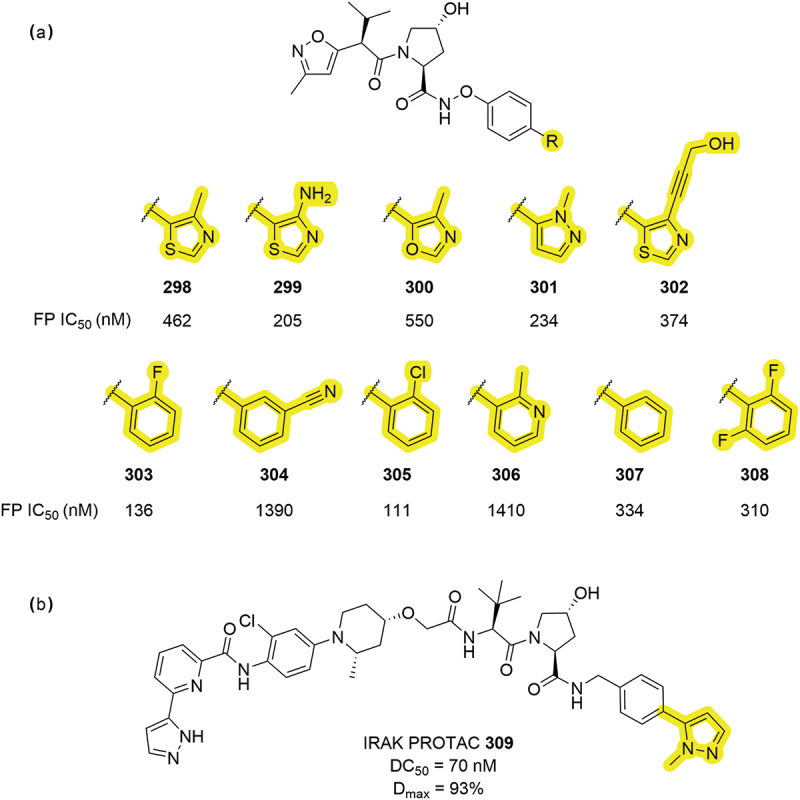
Early attempts of isosteric replacement of the RHS Me-thiazole group. (a) Series of VHL ligands patented by Genetech with IC_50_ calculated by FP. (b) IRAK degrader patented by Janssen bearing Me-pyrazole functionality.

In 2019, Genentech patented a series of VHL inhibitors with a wide variety of alternative aromatic groups to the Me-thiazole (Figure 48(a)) [86,97]. The reported modifications included substituted thiazoles **299** (IC_50_ = 205 nM) and **302** (IC_50_ = 374 nM), that demonstrated improved VCB affinity when compared to the parent thiazole compound **298** (IC_50_ = 462 nM). Further improvements in VCB affinity were observed with fluoro substituted phenyl group **303** (IC_50_ = 136 nM) and the chloro substituted **305** (IC_50_ = 111 nM). Other heterocycles were assessed including isoxazole **300** (IC_50_ = 550 nM) and methyl pyrazole **301** (IC_50_ = 234 nM). The methyl pyrazole group has seen extensive use in PROTACs patented by Janssen in 2021, where degraders of IRAK1 were disclosed showing D_max_ >90% and PROTAC **309**, in particular, returned a 70 nM DC_50_ (Figure 48(b)) [87]. Further PROTAC patents have identified the methyl pyrazole moiety as a useful functional group, having been incorporated into several KRAS PROTACs by Jingrui Biopharma Co. and by Arvinas for SMARCA degraders [92,98].

**Figure 49.**
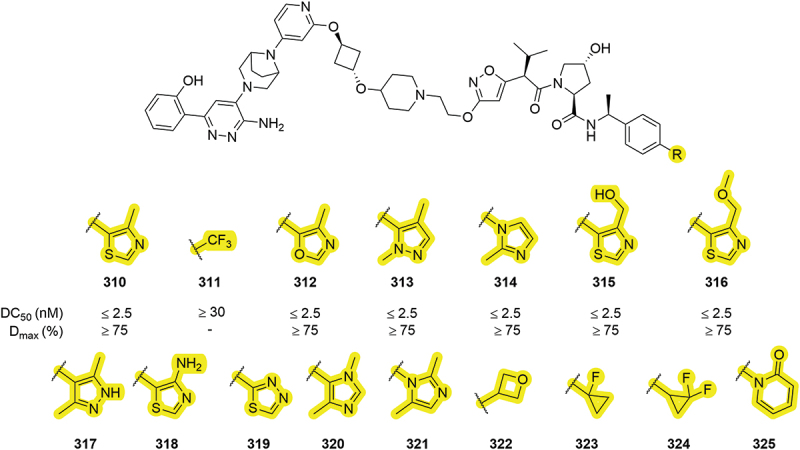
SMARCA degrading PROTACs patented by Arvinas bearing modification to the RHS thiazole.

Work in 2019 by Arvinas disclosed a range of VHL-based PROTACs that degrade SMARCA2/4 proteins which included a range of different heterocycles (Figure 49) [48]. Heterocycles **312-316** were all able to degrade SMARCA2 with a DC_50_ <2.5 nM and D_max_ >75% maintaining the degradation profile to the initial thiazole compound **310**. In addition to heterocycles, some carbocycle groups were covered including cyclopropyl derivatives **323**, **324** and oxetane **322**. Although no degradation data was disclosed for compounds **317-325**, the inclusion of pyridone **325** was an intriguing modification. Pyrazole and imidazole analogues of thiazole (as in **314** and **317**) were subsequently patented in a series of SMARCA2 PROTACs by Kymera in 2022 [57].

**Figure 50.**
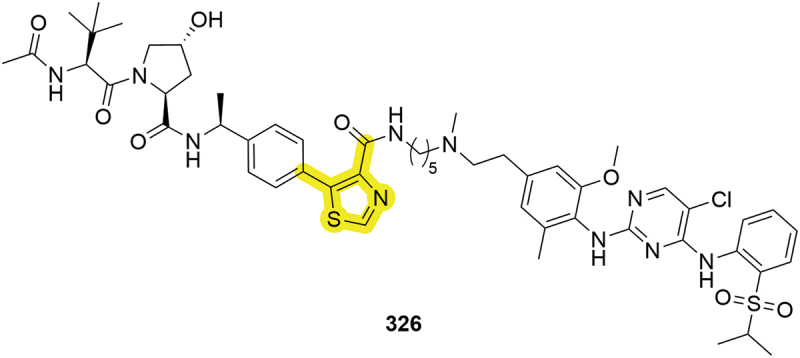
Thiazole-linked **326** targeting protein kinases by Dana Farber Cancer Institute.

In the wake of extended groups from the thiazole moiety being reported (**302**, **315** and **316**), the conjugation of PROTAC linkers from the RHS thiazole was first reported in a patent by the Dana-Farber Cancer Institute in 2021 (**326**, Figure 50) [99]. Although no degradation data was disclosed for compound **326**, subsequent adoption of the thiazole exit vector by others has validated this approach as a *bona fide* strategy within VHL degrader space [29].

**Figure 51.**
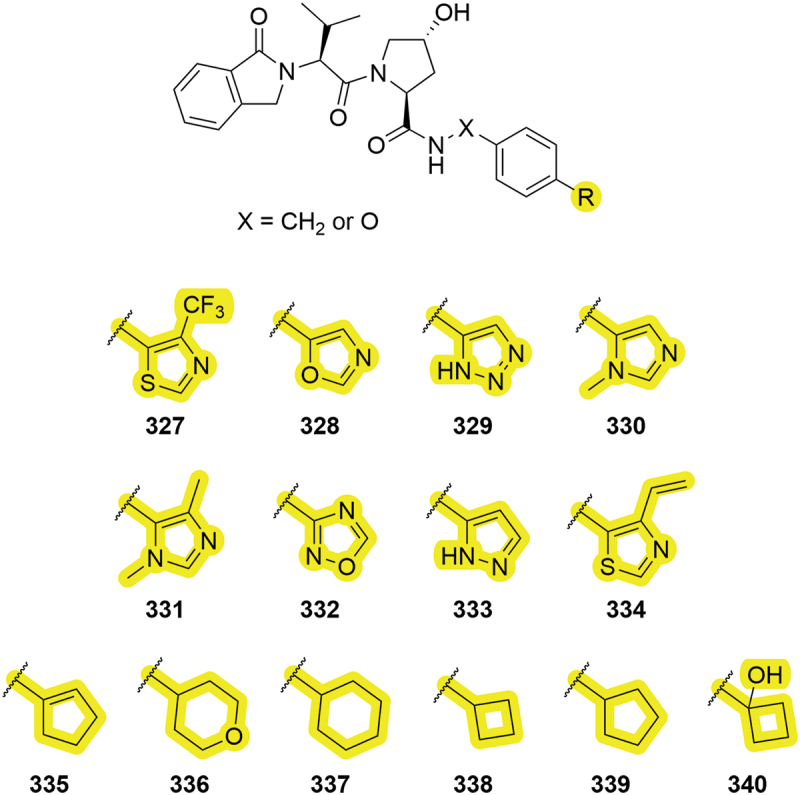
VHL ligands patented by Arvinas in 2019 and 2020 containing various new heterocyclic and cycloalkane modifications.

In further patents from Arvinas in 2019 and 2020, different heterocycles were covered including triazole **329** and oxadiazole **332** for VHL ligands (Figure 51) [49,83]. In addition to heterocycles, new cycloalkyl groups were covered in the patents including 4-tetrahydropyranyl (THP) group **336** and a substituted cyclobutyl ring **340**. These modifications have also been covered in patents discussing PROTACs that degrade Bruton’s tyrosine kinase (BTK) and KRAS proteins [50,100].

**Figure 52.**
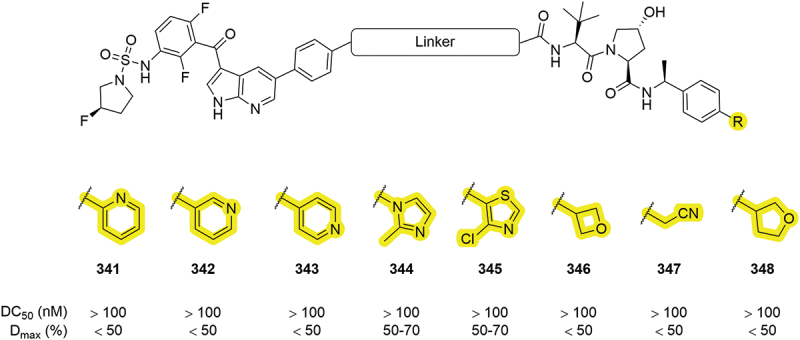
BRAF PROTACs patented by Arvinas containing several changes to the methyl thiazole region.

Arvinas again patented a series of degraders for BRAF proteins in 2020 and here further heterocycles were covered (Figure 52) [52]. Three different pyridyl moieties incorporated into the VHL binding group (**341-343**) performed modestly with all three showing less than 50% D_max_. Two five-membered heterocycles outperformed the pyridyl groups with 50–70% D_max_ values, including the chloro substituted thiazole **345**. Three other alkyl-based groups, **346-348**, showed disappointing D_max_ values < 50%. The alkyl modifications discussed highlight that there are some groups that will not be tolerated in the RHS position.

**Figure 53.**
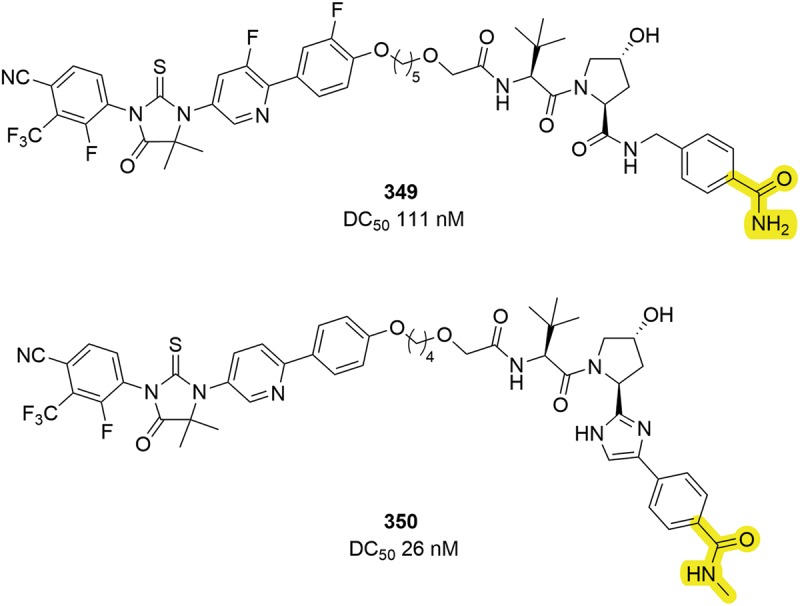
AR degraders containing amide group at the RHS of VHL binding group from Suzhou Kintor.

AR is another protein that has received significant attention in the PROTAC field [101]. Work by Suzhou Kintor in 2023 demonstrated that inclusion of an amide moiety in place of a thiazole can lead to potent AR degraders (Figure 53) [84]. Primary amide **349** showed impressive degradation of AR with a DC_50_ of 111 nM. A secondary amide compound **350**, in combination with an imidazole mimetic discussed in section 4, also demonstrated robust degradation (DC_50_ = 26 nM) indicating that amides can be tolerated in this position with different PROTAC species.

**Figure 54.**
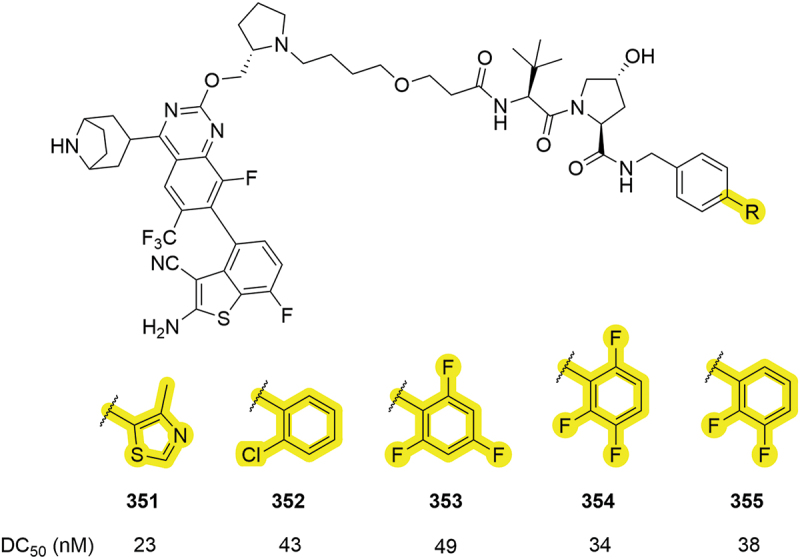
Halo-phenyl containing KRAS PROTACs patented by Erasca.

In 2022, Erasca developed a palette of degraders for KRAS (Figure 54) [56]. Among these PROTACs were three different fluorinated phenyl rings, **353-355**, that all returned DC_50_ values below 50 nM and comparable to the parent thiazole compound **351**. In addition to fluoro-phenyls, a chloro-phenyl group, **352**, returned another potent PROTAC with a 43 nM DC_50_. Substituted phenyl rings are again demonstrated to be tolerated, offering alternatives to other aryl groups.

**Figure 55.**
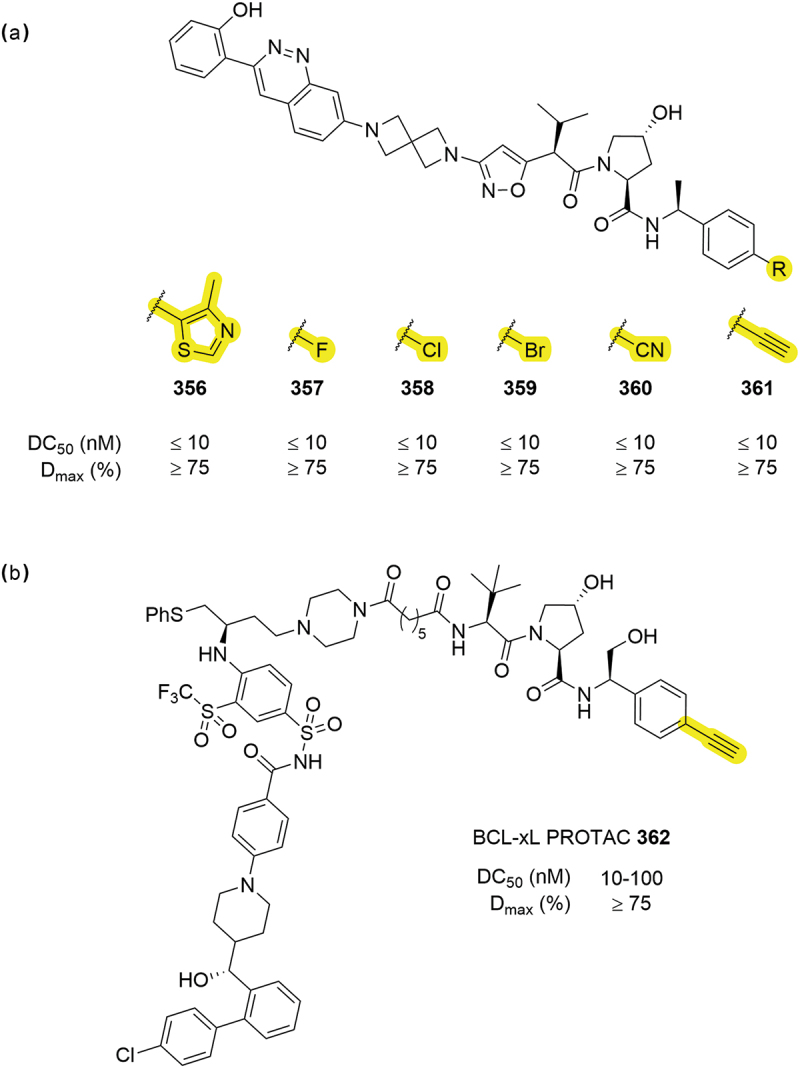
Small group replacements of Me-thiazole. (a) SMARCA2 PROTACs from Foghorn therapeutics containing smaller groups in place of the thiazole. (b) BCL-xL degrader **362** by Kymera containing an alkyne moiety.

Replacement of the Me-thiazole moiety with smaller groups can be advantageous in improving the pharmacokinetic properties of VHL PROTACs, such as reducing molecular weight or the number of hydrogen bond acceptors. Several small atom changes were patented in the context of SMARCA2/4 degraders by Foghorn Therapeutics (Figure 55(a)) [70]. The degraders included three halogen replacements (**357-359**) that were all capable of degrading within the same range as the original thiazole compound **356**. Furthermore, two triple-bond groups in nitrile **360** and alkyne **361** also demonstrated good degrader potential with DC_50_ values <10 nM and D_max_ >75%. Similar small changes were also patented by Ciulli *et al*. for LRRK2 degraders and by Kymera for BCL-xL including alkyne **362** (Figure 55(b)) [79,81,91].

**Figure 56.**
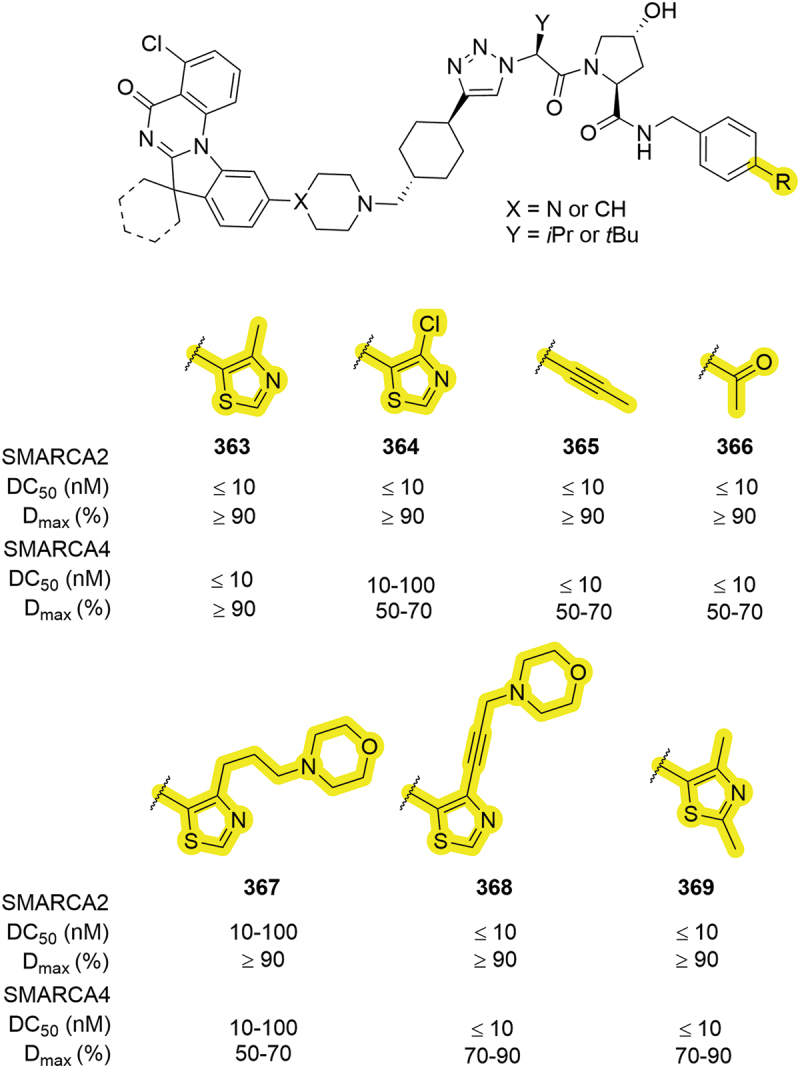
Thiazole modifications developed by the University of Michigan for degrading SMARCA2 and SMARCA4.

In 2024, another series of SMARCA2/4 degraders were developed by the University of Michigan (Figure 56) [85]. A chloro-substituted thiazole, **364**, similar to BRAF degraders by Arvinas, was tolerated with a DC_50_ <10 nM and D_max_ >90%. Further small groups were covered including methyl alkyne **365** and ketone **366**, both achieved maximal degradation of SMARCA2 though lower degradation of SMARCA4 with D_max_ 50–70%. Further interesting functionalisation of the thiazole can be tolerated such as morpholine derivative **367** and **368** as both achieve 90% degradation of SMARCA2, though alkyl linked **368** had a lower D_max_ of 50–70% for SMARCA4. Finally, dimethylated thiazole **369** proved to be a potent degrader of both SMARCA2 and SMARCA4, possessing DC_50_ values below 10 nM for both proteins. The modifications here highlight further the chemical space that can be tolerated around the Me-thiazole region of VHL binding PROTACs with significantly larger groups incorporated but still being potent PROTACs.

The incorporation of the methyl thiazole group remains a staple of VHL ligand design but more and more literature, as shown in this section, indicates that this region of the VHL ligand can be modulated extensively. Ranging from single atoms to new heterocycles to use as an exit vector, the RHS thiazole region of VHL ligands is now becoming significantly more explored in both drug discovery and academic contexts.

## 7. Hydroxyproline modifications and prodrugs strategies

The Hyp core of the VHL ligand is vital for engagement of VHL with high binding specificity, by mimicking the hydroxylated proline of HIF species. Early pioneering work from the Ciulli Lab established 3-fluoro-4-hydroxyprolines as bioisosteric replacements of Hyp, with a single hydrogen at the C-3 position stereoselectively replaced by a fluorine atom [102]. Beyond this study, little success has been attained from attempting to introduce other chemical modifications in the Hyp ring. Due to the essential nature of the Hyp core, only limited examples have thus been published or patented that have tried to either mask or mimic this moiety.

**Figure 57.**
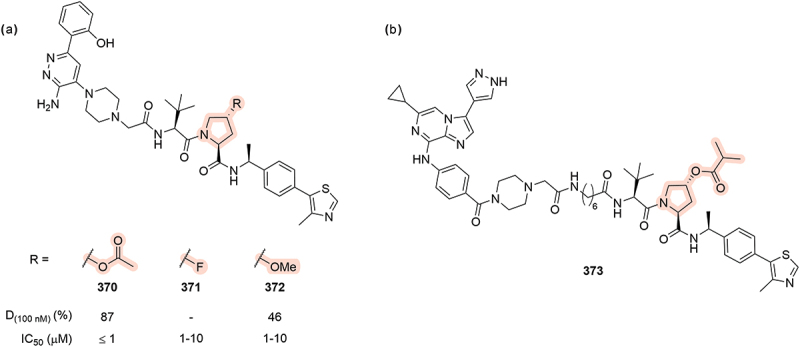
Ester pro-drug masking of hydroxyproline. (a) PROTACs with modified Hyp groups that degrade SMARCA2/4 reported by Aurigene in 2019. D_(100 nM)_ = degradation of SMARCA2 achieved after compound treatment at 100 nM. ‘-’ indicates data not reported. (b) PTK6 targeting PROTAC **373**.

Work covered in a patent by Aurigene highlighted that the hydroxyl group can be masked with an acetyl group, **370** (Figure 57(a)) and still return good degradation of SMARCA2 (D = 87% at 100 nM), presumably following intracellular hydrolysis to reveal the cleaved hydroxyl species. Acetylated **370** also demonstrated anti-proliferative activity in NCI-H929 cells (IC_50_ ≤1 µM). Interestingly, replacement of hydroxyl to a fluorine, **371**, was still able to show some weak anti-proliferative activity (IC_50_ = 1–10 µM). Finally, incorporation of a methoxy moiety, **372**, was still able to degrade SMARCA2 and SMARCA4 (SMARCA2 D = 46% at 100 nM) [71]. The introduction of an acetyl group to mask the Hyp core was also patented for various other PROTAC species including degraders for BCL-xL by Treeline in 2023 [73]. A similar isopropyl ester (**373**) was also patented by the Icahn School of Medicine in 2020 for a protein-tyrosine kinase 6 (PTK6) targeting PROTAC (Figure 57(b)) [103].

**Figure 58.**
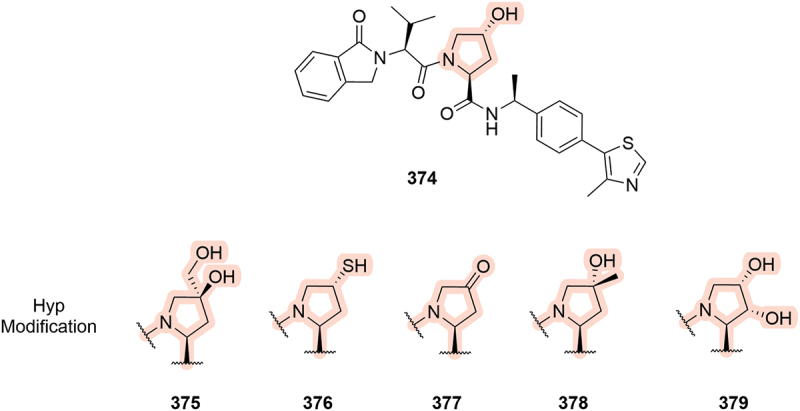
Ligands patented by Arvinas in 2019 bearing modifications to the Hyp core.

In the same year, Arvinas patented a selection of VHL ligands bearing further changes to the Hyp core (Figure 58) [49]. In compound **375**, the canonical (4*R*)-hydroxyl was replaced with a hydroxymethyl group, and the (4*S*)-position was substituted with a second hydroxyl group, equivalent to *cis*-Hyp. Other notable changes to the Hyp core included replacement of the hydroxyl with a (4*R*)-thiol (**376**), oxidation to the corresponding ketone (**377**), and the addition of groups such as (4*S*)-Me (**378**) and (3*R*)-hydroxyl (**379**). Unfortunately, no biological data was published on these compounds so it is unclear how these core modifications may affect binding affinity to VCB.

**Figure 59.**
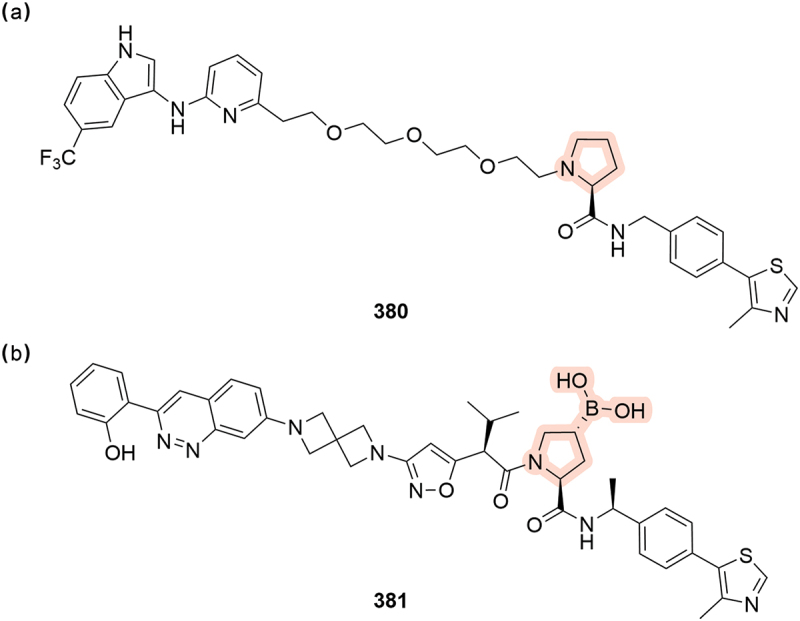
Proline modifications. (a) Putative STING binding PROTAC **380** without the hydroxy proline group from IFMDUE Inc. (b) Boronic acid-bearing PROTAC **381** patented by Foghorn therapeutics.

A patent from IFMDUE Inc. in 2024 looked at degraders of the stimulator of interferon genes protein (STING) and covered interesting modifications of the Hyp core, including removal of the hydroxyl motif completely (**380**, Figure 59(a)) [104]. Removal of the hydroxyl group would seem counterproductive to inducing degradation and no biological assessment of the compounds is provided so it remains unclear if this modification would be tolerated. A further alteration to the Hyp moiety was patented by Foghorn Therapeutics where a boronic acid replaces the hydroxyl group (**381**, Figure 59(b)). The boronic acid likely acts as a covalent PROTAC that binds to VHL and was proposed to modulate BAF complexes [70].

**Figure 60.**
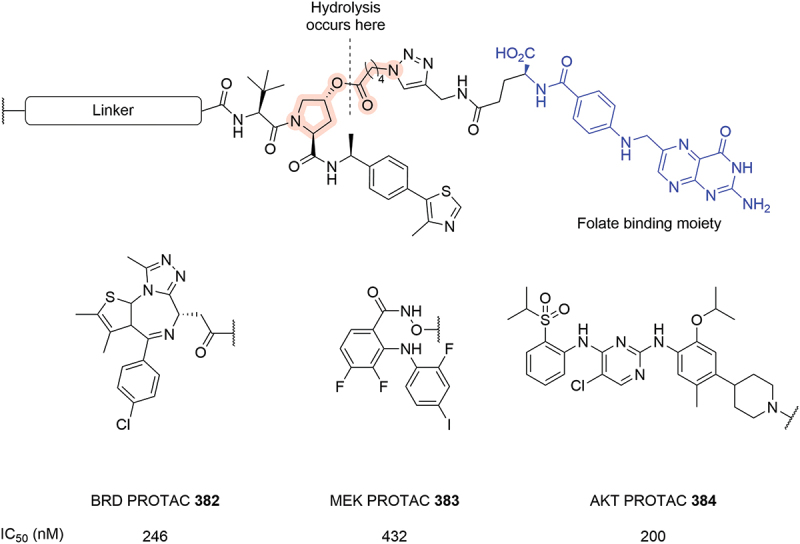
Folate caged PROTACs from BIDMC targeting BRD, MEK and AKT proteins respectively.

The prodrug approach to PROTACs and VHL ligands has seen several patents disclosed since 2019, including work also published by the Beth Israel Deaconess Medical Center Inc. (BIDMC) where folate conjugated molecules are used to selectively target cancer tissue (Figure 60) [105]. The folate group selectively binds to the folate receptor (FOLR-1) on the cell surface and then triggers cellular internalization by endocytosis. The ester linkage is then hydrolyzed intracellularly to release the active PROTAC degrader. The patent shows three different PROTACs that degrade target proteins in a FOLR-1-dependent manner and that each compound caused cell death in several cancer cell lines with reasonable potency (**382** IC_50_ = 246 nM, **383** IC_50_ = 432 nM, **384** IC_50_ = 200 nM) [106].

**Figure 61.**
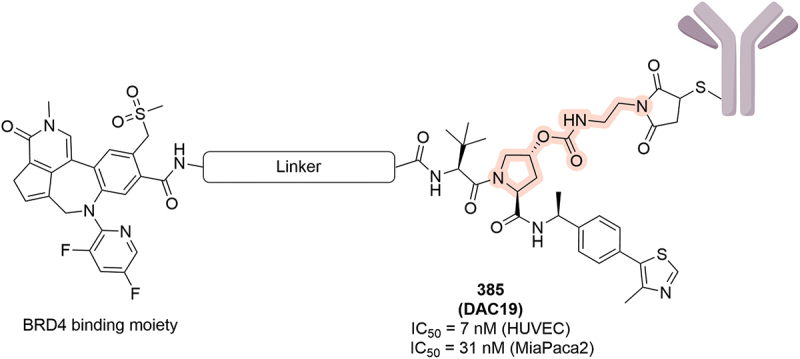
Hydroxyproline modifications exploited in antibody-degrader conjugates (DACs).

Another cell targeting approach was using anti-body drug conjugates (ADCs) to develop degrader antibody conjugates (DACs). In a patent from Anginex Inc., a series of BRD4 DACs conjugated to TM4SF1 antibody were disclosed (Figure 61) [107]. The DACs had effective biological impact, including compound **385** (**DAC19**) that was able to degrade and induce cell death in both HUVEC and MiaPACA2 cell lines (IC_50_ = 7 nM and 31 nM). In the DAC species presented, a carbamate linkage is used to link to the Hyp moiety of the VHL ligand, which is hydrolyzed to release the free PROTAC once the DAC is internalized in the cell [108].

**Figure 62.**
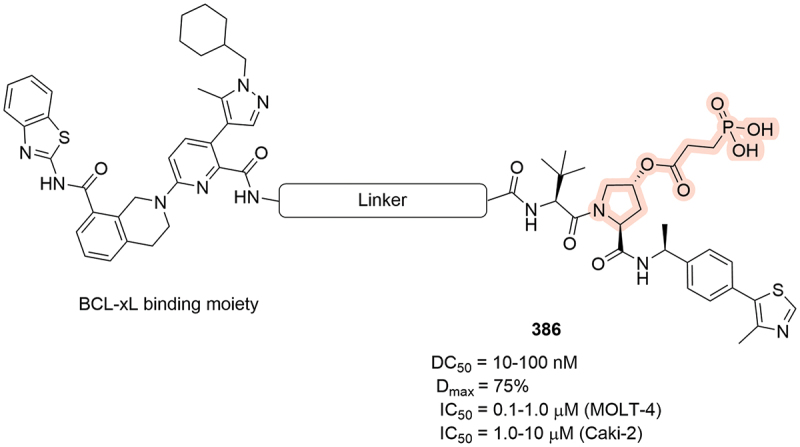
Phosphonate conjugated BCL-xL **386** patented by Kymera.

The prodrug approach was also employed by Kymera to target BCL-xL with phosphonate compound **386** (Figure 62). Prodrug **386** was able to degrade BCL-xL in MOLT-4 tissue with DC_50_ = 10–100 nM and D_max_ = 75%. Further results for **386** demonstrated its ability to induce cancer cell death in both MOLT-4 (IC_50_ = 0.1–1.0 µM) and Caki-2 cells (IC_50_ = 1.0–10 µM) [79].

**Figure 63.**
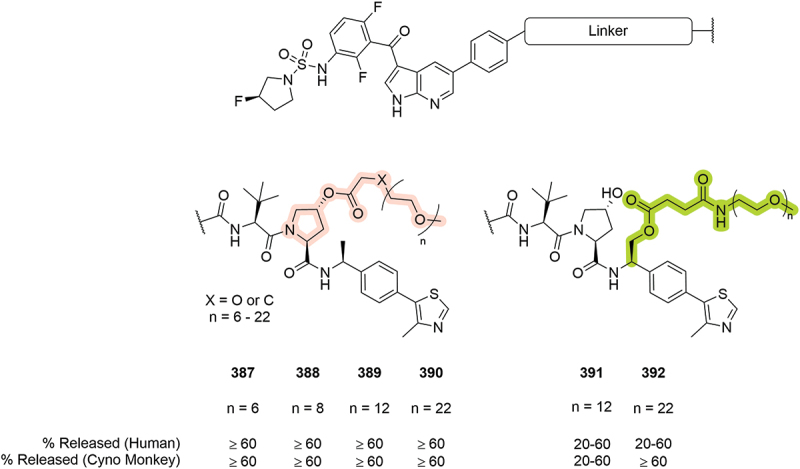
Selected BRAF degrader prodrugs from Arvinas containing PEG solubility handles.

A further example of the prodrug approach was included in a patent by Arvinas where BRAF targeting PROTACs were synthesized with varying PEG units attached to either the Hyp or benzylic positions (Figure 63). The PEG units are incorporated to enhance solubility and are attached via an ester linkage that can be hydrolyzed to release the active PROTAC [52]. The % released of free PROTAC was calculated in both human and cyno monkey plasma. In general, examples linked from the Hyp moiety (**387-390**) showed over 60% release in both human and monkey. Exemplar benzylic linked prodrugs (**391** and **392**), however, showed lower release in in human plasma. This prodrug approach underlines the possibility to functionalize various parts of the VHL ligand and still deliver the required PROTAC payload to ensure a therapeutic effect.

Despite the apparent necessity for the Hyp core to engage VHL, there are several patents that indicate this can be modified although not as significantly as other sections of the VHL ligand. The ability to make PROTAC prodrugs, however, is becoming more established, offering the ability to target specific tissues and aid cellular uptake of PROTACs.

## 8. Expert opinion

The rise of VHL patent literature in the last 5 years aptly reflects the growing clinical and commercial interest in targeted protein degradation as an emergent therapeutic modality. As frontrunner degrader clinical assets, such as **ARV-471** (Vepdegestrant) and Nurix **NX-5948**, are now either entering phase III clinical trials or are primed for an accelerated clinical path, the biotechnology markets and healthcare providers eagerly await the first FDA-approved PROTAC degraders.

Although Cereblon-based degraders account for the majority of PROTACs currently in clinical development, there is still considerable interest in VHL-based degraders. The acceleration in the growth of patents covering VHL-based compounds shows that there is significant investment being undertaken to develop clinically relevant VHL-based degraders as molecular therapeutics.

The type of new modifications that are made to VHL ligands reflects the current challenges faced in the development of orally bioavailable and efficacious VHL-based degraders; namely, high lipophilicity, high molecular weight and the presence of essential amide bonds and hydrogen-bond donors.

The LHS amide has seen the emergence of heterocyclic replacements, effectively removing the hydrogen bond donor that contributes to the poor overall permeability of many VHL-based degraders. Among these modifications, the isoxazole and 1,2,3-triazole have found the most wide-spread use in current patent literature and this trend is likely to continue in coming years.

Interest in alternatives to the *tert*-leucine moiety has seen a growth in recent years, with most modifications being investigated to move away from the peptidic nature while retaining binding affinity and modulating polarity. Whilst expansion in this region is generally tolerated, it is unlikely to add any meaningful changes to binding affinity due to its trajectory out of the VHL binding pocket. On the other hand, exploitation of this solvent-exposed nature has led to developments in PROTACs linked via this vector.

The RHS amide region has been modified in various ways to improve the ‘drug-like’ properties of the ligands, and subsequently the PROTACs. Several attempts have been made to replace or mask the H-bond donor, such as introducing isosteres of the amide group – the mimetics being extremely successful in some cases. Due to its solvent-exposed nature, the benzylic position has been used as a handle for linker attachment and several solubilizing groups with different heteroatoms have been introduced, as well as cyclized versions onto the phenyl ring. These modifications have provided mixed results, sometimes proving beneficial and sometimes detrimental to the compounds degradation profile.

The phenyl ring moiety has also received attention in the recent years, with the introduction of different types of substituents as well as its replacement with heteroaromatic or aliphatic rings. These changes could enhance the physiochemical properties of the PROTACs and in some cases, improve their degradation potential.

The methyl-thiazole group has been modified in a variety of ways, again highlighting the adaptability of VHL ligands. Good affinity to VHL can be achieved when replacing this group with both small atoms and significantly larger and flexible species. The untapped potential of linkerology in this space could also lead to a new exciting series of PROTACs that further enhance the TPD field. Modification of the thiazole can, in certain cases, lead to loss of VHL affinity, although this can often be recouped from other modifications elsewhere in the VHL ligand.

The Hyp motif remains the most conserved and unchanged part of the VHL ligand. Efforts to replace or mimic this key group have been met with limited success and to date the group remains untouched in the vast majority of VHL chemical space. The Hyp group can be masked in an attempt to improve properties or incorporated into prodrug-like space via derivatization of its hydroxyl group with cellular labile bonds. Efficient release of the hydroxy group remains essential to achieve good levels of binding and cellular activity.

The adaptability of VHL-based inhibitors and PROTACs covered in patents indicates that an expanded chemical space is expected to be covered in the near future. Techniques that are also on the rise could be coupled with VHL ligand development to rapidly accelerate its therapeutic potential. For example, ‘direct-to-biology’ (D2B) approaches could help to identify functional groups in each part of the VHL ligand that are permissive of good degradation activity in a high-throughput manner [109,110]. Recent advances made with CryoEM to resolve ternary complex structures at increasingly higher resolution and to understand the mechanism of VHL mediated PROTAC degradation and ubiquitination could also allow for new insights into developing VHL-based degraders [111,112].

Although some exciting modifications have been disclosed, much of the recent patent literature still uses the canonical **VH032** scaffold and LHS amide exit vector. Modifications of note include the introduction of amide mimetics that can remove the peptidic nature of the VHL scaffold and the use of the RHS thiazole group as an exit vector for linkers, both offering great potential to design new degraders. Another key modification is the use of a cycloheptyl ring in **GNE7599**, which to our knowledge, is the most potent VHL inhibitor reported to date but has not yet been utilized for targeted protein degradation. Overall, the VHL chemical space continues to grow, and it is expected that there will soon be more VHL-based compounds reaching the clinic thanks to the progress established in the patent literature as reviewed to date.

## Article highlights

- This review covers the novel structural changes to the VHL core scaffold described in patents disclosed between January 2019 and August 2024.
- VHL is an E3 ubiquitin ligase and tumor suppressor that regulates HIF-alpha substrates by triggering their ubiquitination and proteasomal degradation.
- The ability to ‘hijack’ VHL E3 catalytic activity with small-molecules has led to the rise of VHL-based heterobifunctional degraders (PROTACs).
- VHL ligand and PROTAC development has been progressed by structure-guided design using ligand-bound co-crystal structures of VHL.
- Currently, there are 4 VHL-based PROTACs undergoing clinical trials (**DT2216**, **KT-333**, **ASP3082**, **PRT3789**).
- There is growing clinical interest resulting in rapid chemical innovation of VHL binding small molecules.
